# Supplementary material for: Effect of an editorial intervention to improve the completeness of reporting of randomised trials: a randomised controlled trial
Source: BMJ Open. 2020 May 18;10(5):e036799. doi: 10.1136/bmjopen-2020-036799 (PMC7239541; doi:10.1136/bmjopen-2020-036799)
Supplement: Supplementary data [file bmjopen-2020-036799supp001.pdf]

<u>N/As for a certain item</u>

For items reported as N/A in the CONSORT checklist, we consider them as:

- Adequately reported if (i) the item did not apply and therefore it did not have to be reported, and (ii) the item applied and it was actually reported although the page number was not given.
- Inadequately reported if the item did apply but it was not adequately reported.

<u>Rules about specific items:</u>

- **Item 8a** ("*Method used to generate the random allocation sequence*"): inadequately reported if authors have reported this information elsewhere but not in the main body of the article. According to CONSORT, "*it is important that information on the process of randomisation is included in the body of the main article and not as a separate supplementary file; where it can be missed by the reader*".
- **Item 11a** ("*If done, who was blinded after assignment to interventions (for example, participants, care providers, those assessing outcomes) and how*"): adequately reported if blinding was not performed and authors explicitly said so, and inadequately reported if blinding was assumed to be not performed and authors did not mention it in the manuscript.
- **Item 13a** ("*For each group, the numbers of participants who were randomly assigned, received intended treatment and were analysed for the primary outcome*") and **item 13b** ("*For each group, losses and exclusions after randomisation, together with reasons*")**:** the corresponding information could be included either in the text or in the flow diagram. If information was only included in the discussion, it was considered as inadequately reported.
- **Item 17a** ("*For each primary and secondary outcome, results for each group, and the estimated effect size and its precision (such as 95% confidence interval)*")**:** adequately reported if there was a correspondence between the outcomes in the results section and the ones listed in the methods section (and therefore evaluated in Item 6a).
- **Extension of Item 17a** for Pilot and Feasibility trials ("*For each objective, results including expressions of uncertainty (such as 95% confidence interval) for any estimates. If relevant, these results should be by randomised group*"): we did not expect authors to

report the effect sizes but the results (plus expressions of uncertainty) for each objective.
