# Supplementary material for: Effect of an editorial intervention to improve the completeness of reporting of randomised trials: a randomised controlled trial
Source: BMJ Open. 2020 May 18;10(5):e036799. doi: 10.1136/bmjopen-2020-036799 (PMC7239541; doi:10.1136/bmjopen-2020-036799)
Supplement: Supplementary data [file bmjopen-2020-036799supp002.pdf]

We divided the 24 included manuscripts into 4 batches of 6 manuscripts.

Every time DB detected in the submissions report (see "Preliminary work" section) that all 6 manuscripts of each batch had been revised by authors, he first made available to the outcome assessors the submitted version of the manuscript (version 1). Assessors had to complete the evaluation form for each manuscript independently and in duplicate. This form included the CONSORT extensions to be used. Assessors could explicitly indicate in it that they wanted to discuss a specific item with the other assessor. Once they were done with all manuscripts' version 1, DB informed them of the discrepancies between their evaluations, which were resolved by consensus. Afterwards, he shared the manuscript revised by the authors (version 2) and we repeated the outcome evaluation process.

This process was done for the 4 batches of 6 manuscripts.
