# Supplementary material for: Effect of an editorial intervention to improve the completeness of reporting of randomised trials: a randomised controlled trial
Source: BMJ Open. 2020 May 18;10(5):e036799. doi: 10.1136/bmjopen-2020-036799 (PMC7239541; doi:10.1136/bmjopen-2020-036799)
Supplement: Supplementary data [file bmjopen-2020-036799supp003.pdf]

Script A: Power calculation

```
na <- 12
nb <- 12
A <- 0:8
B <- 0:8
#Estimated probabilities of reporting 0,...,8 items in the intervention group (a) and control group (b)
pra <- c(0, 0, 0, 0, 0, 0, 0, 5, 5)
pra <- pra/sum(pra)
prb <- c(0, 0, 0, 0, 0, 2, 4, 4, 2)
prb <- prb/sum(prb)
nboot <- 1000
N <- 1000
#Matrices containing random samples of scores for the two groups
ma <- matrix(sample(A, pr = pra, replace = TRUE, siz = N * na), ncol = N)
mb <- matrix(sample(B, pr = prb, replace = TRUE, siz = N * nb), ncol = N)
#Generation of N confidence intervals
sign1 <- c()
for (i in 1:N) {
  reporting <- data.frame(
    score = c(ma[, i], mb[, i]),
    group = factor(rep(c("Control", "Intervention"), c(na, nb)))
  )
  # Bootstrapping
  diff.mean1 <- c()
  for (k in 1:nboot){
    sel <- sample(1:(na + nb), na + nb, rep = TRUE)  # selected articles
    reporting.boot <- reporting[sel, ]
    diff.mean1[k]<- with(reporting.boot, diff(tapply(score, group, mean)))
  }
  conf.int1 <- quantile(diff.mean1, c(0.025, 0.975))
  sign1[i] <- conf.int1[2] < 0 #Checking if the CI crosses 0
}
power <- sum(sign1)/N
```

Script B: Randomisation of manuscripts (R Shiny application)

```
#File 1
Sys.setlocale("LC_ALL", "es_ES.UTF-8") #to be sure that accents in text will be allowed in plots
library(shiny)
library(shinyalert)
fluidPage(
  useShinyalert(),
  fluidRow(
        headerPanel('Randomisation of BMJ Open manuscripts'),
        wellPanel(
                textInput("identif", "Please enter the manuscript ID:", width='33%'),
                textAreaInput("titulo", "Please enter the manuscript title, or at least its first words:"),
                selectInput("tipo", "Does it correspond to an extension of CONSORT?", choices=c("", "Yes", "No"), width='33%'),
                actionButton("send", "SUBMIT")
        ),
        p() ,
        wellPanel(
                #h3('Data'),
                #p("(just for testing purpose)"),
                #tableOutput("asig"),
                #actionButton("borrar", "RESET")
        )
  )
```

```
#File 2
library(shiny)
library(shinyalert)
library(blockrand)
is.void = function(x) {
   if (is.null(x)) return(TRUE)
         x == ''
}
shinyServer(function(input, output, session) {
upda = reactiveValues(asg=NA)
archi = "asignacion.dat"
```

```
#File 2 (continuation)
if (file.exists(archi)) {
        upda$asg = read.table(archi, header=TRUE, sep='\t', stringsAsFactors=FALSE)
} else {
   upda$asg = data.frame()
}
set.seed(6374422)
ext <- blockrand(n=100, id.prefix='E', block.prefix='B',stratum='Extension', block.sizes=c(2,2))
noext <- blockrand(n=100, id.prefix='N', block.prefix='B',stratum='No Ext.', block.sizes=c(2,2))
  go <- eventReactive(input$send, {
   list(id=input$identif, tit=input$titulo, ex=input$tipo)
  })
block_random = function(tip) {
  if (dim(upda$asg)[1]>0) {
    X = subset(upda$asg, strat==tip)
        x = dim(X)[1]
  } else x = 0
  if (tip=="Yes") {
                g = ext$treatment[x+1]
        } else if (tip=="No") {
                g = noext$treatment[x+1]
        } else return(-1)    # Error
  ifelse(g=='A', 0, 1)
}
        observeEvent(input$send, {
                Q = go()
                if (is.null(Q) | is.void(Q)) {
                        shinyalert(title="Please fill in the input.", type="error", showConfirmButton=TRUE, confirmButtonText="OK", timer=0)
                        return()
                }
                id = Q$id
                check.id = grep("^bmjopen-201[89]-[0-9]{6}$", id)
                if (length(check.id) == 0) {
                        shinyalert(title="Wrong ID.", text="Please enter a valid BMJ code.", type="error", showConfirmButton=TRUE, confirmButtonText="OK", timer=0)
return()
                }
```

```
#File 2 (continuation)

id = Q$id

        check.id = grep("^bmjopen-201[89]-[0-9]{6}$", id)

        if (length(check.id) == 0) {

            shinyalert(title="Wrong ID.", text="Please enter a valid BMJ code.", type="error", showConfirmButton=TRUE, confirmButtonText="OK", timer=0)

            return()

        }

        if (Q$ex=='') {

            shinyalert(title="Empty field:", text="extension of CONSORT?", type="error", showConfirmButton=TRUE, confirmButtonText="OK", timer=0)

            return()

        }

        if (Q$tit=='') {

            shinyalert(title="Empty field:", text="please provide a title.", type="error", showConfirmButton=TRUE, confirmButtonText="OK", timer=0)

            return()

        }

        n = dim(upda$asg)[1]

        if (n>0) {

            I = which(upda$asg$id==id)

            if (length(I)>0) {

                shinyalert(title="Invalid ID:", text="this ID has been already assigned.", type="error", showConfirmButton=TRUE, confirmButtonText="OK", timer=0)

                return()

            }

        }

        txt = paste("Go on with the manuscript '<i>", Q$tit, "</i>', with ID <b>",Q$id, "</b>, which <b>", ifelse(Q$ex=='Yes', 'corresponds', 'does not correspond'), "</b> to an extension of CONSORT:", sep='')

        shinyalert(title='Confirm inclusion', text=txt, closeOnEsc=TRUE, closeOnClickOutside=FALSE, html=TRUE, type="warning", showConfirmButton=TRUE, showCancelButton=TRUE, confirmButtonText="Right, go on", cancelButtonText="NO, stop", timer=0, imageUrl="", callbackR = Success)

    })

    Success = function(x) if (x != FALSE) {

        Q = go()

        g = block_random(Q$ex)

        if (g == -1) return

        L = list(id=Q$id, title=Q$tit, strat=Q$ex, group=g, date=date())

        upda$asg = rbind(upda$asg, as.data.frame(L))

        write.table(upda$asg, archi, sep='\t', row.names=FALSE)
```

```
#File 2 (continuation)
            filename = tempfile()
            interv = ifelse(g==0, "CONTROL (0)", "INTERVENTION (1)")
            cat(sprintf("Manuscript ID: %s\nTitle: %s\nExtension of CONSORT: %s\nAssigned to: %s\n",
                  Q$id, Q$tit, Q$ex, interv), file=filename)
            # preparar y mandar mensaje
            dest = 'david.blanco@hotmail.com'
            Msg = tempfile()
            comm = paste('echo "To:', dest, '\nFrom: jose.a.gonzalez@upc.edu\nSubject: A manuscript has been assigned\n"| (cat -', filename, ') >', Msg)
            system(comm)
            system(paste("ssmtp", dest, "<", Msg))

            updateTextInput(session, "identif", "Please enter the manuscript ID:", value='')
            updateTextAreaInput(session, "titulo", "Please enter the manuscript title, or at least its first words:", value='')
            updateSelectInput(session, "tipo", "Does it correspond to an extension of CONSORT?", choices=c("", "Yes", "No"))
      }

      observeEvent(input$borrar, {
            shinyalert(title='Are you sure?', text="This action will remove the assignations.", closeOnEsc=TRUE, closeOnClickOutside=FALSE, html=TRUE, type="warning", showConfirmButton=TRUE, showCancelButton=TRUE, confirmButtonText="Yes, remove them", cancelButtonText="NO, don't reset", timer=0, imageUrl="", animation=TRUE, callbackR = function(x) { if(x != FALSE) { upda$asg = data.frame(); file.remove(archi) } })
      })
      output$asig = renderTable(  {
            upda$asg
      } )
})
```

Script C: Primary outcome analysis

```
# Loading the data
data <- read.csv2('Scores.txt', header = TRUE, sep = '')
data2 <- subset(data, data$Imputation==0)
# Fitting a linear model and calculating CIs with imputation
model1 <- lm(data$Final ~ data$Baseline + data$Group)
summary(model1)
na <- 12
nb <- 12
nboot <- 10000
set.seed(111111)
diff.mean1 <- c()
for (k in 1:nboot){
  sel <- sample(1:(na + nb), na + nb, rep=TRUE)  # selected articles
  reporting.boot <- data[sel, ]
  diff.mean1[k] <- coefficients(lm(reporting.boot$Final ~ reporting.boot$Group
+ reporting.boot$Baseline, reporting.boot))[2]
}
conf.int1 <- quantile(diff.mean1, c(0.025, 0.975), na.rm = TRUE)
# Fitting a linear model and calculating CIs without imputation
model2 <- lm(data2$Final ~ data2$Baseline + data2$Group)
summary(model2)
na <- 9
nb <- 9
set.seed(222222)
diff.mean2 <- c()
for (k in 1:nboot){
  sel <- sample(1:(na + nb), na + nb, rep = TRUE)  # selected articles
  reporting.boot <- data2[sel, ]
  diff.mean2[k] <- coefficients(lm(reporting.boot$Final ~ reporting.boot$Group
+ reporting.boot$Baseline, reporting.boot))[2]
}
conf.int2 <- quantile(diff.mean2, c(0.025, 0.975), na.rm = TRUE)
```
