# Supplementary figures and images for: Effect of an editorial intervention to improve the completeness of reporting of randomised trials: a randomised controlled trial
Source: BMJ Open. 2020 May 18;10(5):e036799. doi: 10.1136/bmjopen-2020-036799 (PMC7239541; doi:10.1136/bmjopen-2020-036799)

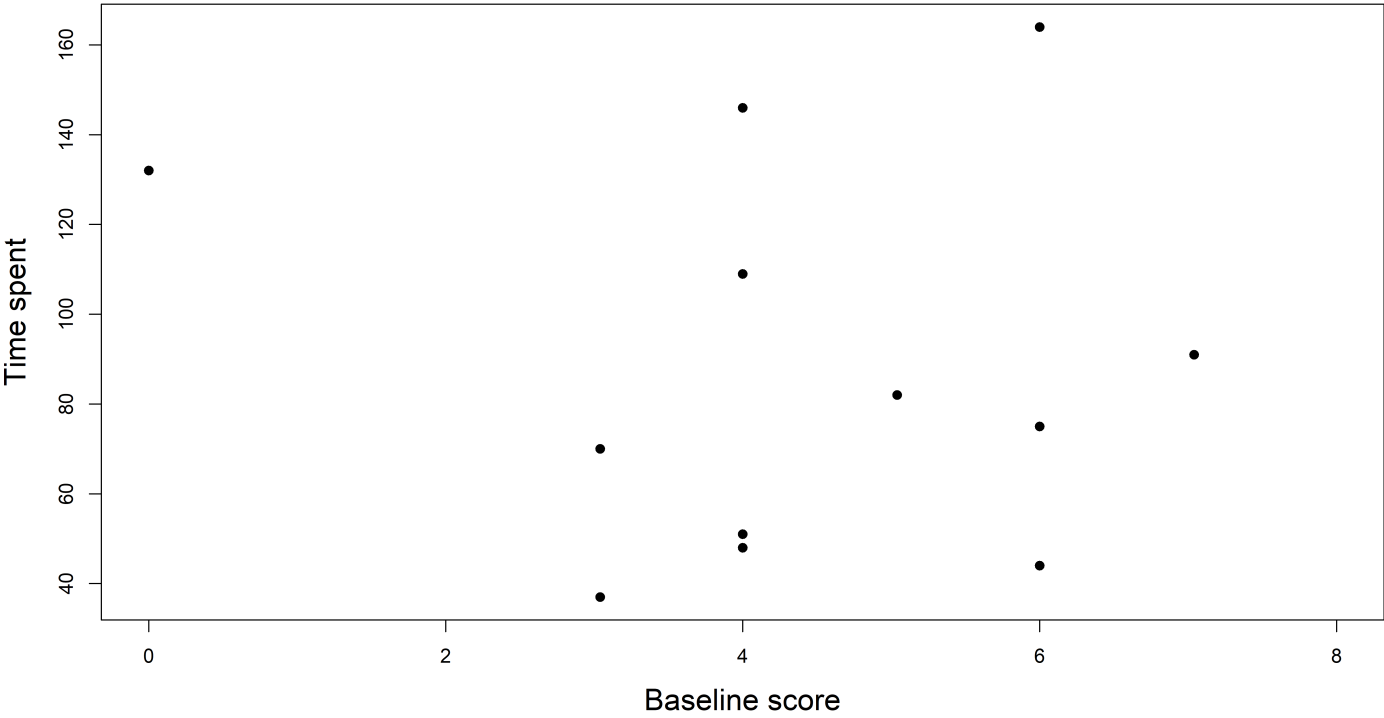

Supplement: Supplementary data [file bmjopen-2020-036799supp004.pdf]
