# Supplementary material for: Effect of an editorial intervention to improve the completeness of reporting of randomised trials: a randomised controlled trial
Source: BMJ Open. 2020 May 18;10(5):e036799. doi: 10.1136/bmjopen-2020-036799 (PMC7239541; doi:10.1136/bmjopen-2020-036799)
Supplement: Author's manuscript [file bmjopen-2020-036799.draft_revisions.pdf]

BMJ Open is committed to open peer review. As part of this commitment we make the peer review history of every article we publish publicly available.

When an article is published we post the peer reviewers' comments and the authors' responses online. We also post the versions of the paper that were used during peer review. These are the versions that the peer review comments apply to.

The versions of the paper that follow are the versions that were submitted during the peer review process. They are not the versions of record or the final published versions. They should not be cited or distributed as the published version of this manuscript.

BMJ Open is an open access journal and the full, final, typeset and author-corrected version of record of the manuscript is available on our site with no access controls, subscription charges or pay-per-view fees (http://bmjopen.bmj.com).

If you have any questions on BMJ Open's open peer review process please email info.bmjopen@bmj.com

Effect of an editorial intervention to improve the completeness of reporting of randomised trials: a randomised controlled trial

Journal:	*BMJ Open*
Manuscript ID	bmjopen-2020-036799
Article Type:	Original research
Date Submitted by the Author:	06-Jan-2020
Complete List of Authors:	Blanco, David; Universitat Politecnica de Catalunya, Statistics and Operations Research Department; Université de Paris, CRESS, INSERM, INRA<br>Schroter, Sara; BMJ Editorial,<br>Aldcroft, Adrian<br>Moher, David; Ottawa Hospital Research Institute, Ottawa Methods Centre<br>Boutron, Isabelle; Université de Paris, CRESS, INSERM, INRA<br>Kirkham, Jamie J.; Manchester University, Centre for Biostatistics, Manchester Academic Health Science Centre<br>Cobo, Erik; Universitat Politecnica Catalunya, Statistics and Operational Research Department
Keywords:	MEDICAL JOURNALISM, STATISTICS & RESEARCH METHODS, MEDICAL EDUCATION & TRAINING

SCHOLARONE™
Manuscripts

I, the Submitting Author has the right to grant and does grant on behalf of all authors of the Work (as defined in the below author licence), an exclusive licence and/or a non-exclusive licence for contributions from authors who are: i) UK Crown employees; ii) where BMJ has agreed a CC-BY licence shall apply, and/or iii) in accordance with the terms applicable for US Federal Government officers or employees acting as part of their official duties; on a worldwide, perpetual, irrevocable, royalty-free basis to BMJ Publishing Group Ltd ("BMJ") its licensees and where the relevant Journal is co-owned by BMJ to the co-owners of the Journal, to publish the Work in this journal and any other BMJ products and to exploit all rights, as set out in our licence.

The Submitting Author accepts and understands that any supply made under these terms is made by BMJ to the Submitting Author unless you are acting as an employee on behalf of your employer or a postgraduate student of an affiliated institution which is paying any applicable article publishing charge ("APC") for Open Access articles. Where the Submitting Author wishes to make the Work available on an Open Access basis (and intends to pay the relevant APC), the terms of reuse of such Open Access shall be governed by a Creative Commons licence – details of these licences and which Creative Commons licence will apply to this Work are set out in our licence referred to above.

Other than as permitted in any relevant BMJ Author's Self Archiving Policies, I confirm this Work has not been accepted for publication elsewhere, is not being considered for publication elsewhere and does not duplicate material already published. I confirm all authors consent to publication of this Work and authorise the granting of this licence.

For peer review only

Effect of an editorial intervention to improve the completeness of reporting of randomised trials: a randomised controlled trial

David Blanco[1, 2] (ORCID: 0000-0003-2961-9328), Sara Schroter[3] (ORCID: 0000-0002-8791-8564), Adrian Aldcroft[3] (ORCID: 0000-0003-0106-720X), David Moher[4] (ORCID: 0000-0003-2434-4206), Isabelle Boutron[2] (ORCID: 0000-0002-5263-6241), Jamie J Kirkham[5**] (ORCID: 0000-0003-2579-9325) and Erik Cobo[1**] (ORCID: 0000-0002-3534-5602).

[1]Statistics and Operations Research Department, Universitat Politècnica de Catalunya, Barcelona, Spain.

[2]Université de Paris, CRESS, INSERM, INRA, Paris F-75004, France.

[3]The BMJ, London, UK.

[4]Centre for Journalology, Ottawa Hospital Research Institute, Ottawa, Canada.

[5]Centre for Biostatistics, Manchester Academic Health Science Centre, University of Manchester, Manchester, UK.

Correspondence to David Blanco; david.blanco.tena@upc.edu

**JJK and EC are joint senior authors.

For peer review only - http://bmjopen.bmj.com/site/about/guidelines.xhtml

Abstract

Objective: To evaluate the impact of an editorial intervention to improve the completeness of reporting of reports of randomised trials.

Design: Randomised controlled trial.

Setting: Peer review process of BMJ Open.

Participants: 24 manuscripts describing randomised controlled trials (RCTs).

Interventions: We used an R Shiny application to randomise manuscripts (1:1 allocation ratio, blocks of 4) to the intervention (n = 12) or the control group (n = 12). The intervention consisted of (i) an evaluation by a CONSORT expert of the consistency between eight core items of the submitted CONSORT checklist and the manuscript, and (ii) the production of a standardised report containing precise requests for authors, which was included in the decision letter to authors alongside the standard peer review reports. Manuscripts in the control group underwent usual peer review. Authors of manuscripts were unaware that they were part of an RCT.

Primary outcome measure: Number of adequately reported items (0 to 8 scale) in the manuscript revised by authors after the first round of peer review. We imputed missing data for the main analysis (n = 24). Secondarily, we considered the complete case analysis (n = 18). Two blinded reviewers assessed outcomes independently and in duplicate. Disagreements among them were solved by consensus.

Results: The manuscripts that received the intervention were more completely reported than the ones that underwent the standard review process: intervention group (mean: 7.01; SD: 1.47), control group (mean: 5.68; SD: 1.43), mean difference 1.43 (95% CI: 0.31 to 2.58). The complete case analysis yielded a difference of 1.75 (95% CI: 0.80 to 2.75).

Conclusions: In this study, we provide empirical evidence of the benefit of involving trial reporting experts in the peer review process. Improving the completeness of RCTs is essential to improve their usability and reduce research waste.

Trial registration: *ClinicalTrials.gov*, Identifier NCT03751878.

For peer review only

Strengths and weaknesses

- We used a randomised trial design and implemented the intervention in a real editorial context.
- Outcome assessment was blinded and in duplicate.
- We focused only on eight items of one reporting guideline (CONSORT).
- The intervention was performed in only one journal.

Introduction

The lack of transparency and accuracy of research reports has been pointed out as one of the main factors causing research waste (1). Adequate reporting allows researchers to replicate results, generate new hypothesis or compare the results of different studies; it allows health care professionals to make clinical decisions; it allows governments to change public policies; and it helps patients to be aware of what healthcare options they have (2).

Reporting guidelines (RGs) are sets of recommendations for authors, usually in the form of a checklist, on how to report research methods and findings so that no relevant information is omitted (2). Since the inception in 1996 of the Consolidated Standards of Reporting Trials (CONSORT) for the reporting of randomised controlled trials (RCTs) (3), hundreds of RGs for different study types, data, and preclinical and clinical areas have been developed (4). CONSORT is currently one of the most well-established RGs and has been revised and updated twice (5,6).

Most RGs have not been evaluated as to whether they actually improve completeness of reporting. Even for those that have been shown to be beneficial, such as CONSORT, the degree of author adherence is poor (7). For this reason, a range of interventions aimed to improve adherence to RGs have been proposed, and the impact of some of these on completeness of reporting have been evaluated. A recent scoping review identified and classified 31 interventions targeting different stakeholders, including authors, peer reviewers,

For peer review only - http://bmjopen.bmj.com/site/about/guidelines.xhtml

journal editors, medical schools and ethics boards (8). Among these, only four were assessed in RCTs and their effects were varied (9–12). Most of the studies included in the scoping review described observational studies that evaluated the pooled effect of different journal strategies, which ranged from (i) making available editorial statements that endorse certain RGs, (ii) recommending or requiring authors to follow RGs in the "Instructions to authors", and (iii) requiring authors to submit a completed RG checklist together with the manuscript. However, these actions have been shown not to have the desired effect (13–16). In contrast, completeness of reporting improved remarkably when editors were in the process of checking adherence to RGs (17).

Recently, many biomedical journals have opted for strategy (iii) above as a way to enforce the use of RG checklists. While sometimes checking these is delegated to unpaid peer reviewers, journal editors generally report that this task goes beyond the role of these and that it may even decrease the quality of peer review reports (18). If checking reporting issues becomes a standard exercise for peer reviewers, some editors are afraid that peer reviewers may be less likely to comment on important aspects of a manuscript, such as its importance, novelty, and relevance. Involving trained experts or administrative staff could be a way to make the most of this editorial strategy (18).

Study objectives

We describe an RCT to evaluate the effect of an editorial intervention performed by a CONSORT expert on the completeness of reporting of trials submitted to BMJ Open, compared to the standard peer review process.

Methods

Trial design and study setting

This was a two-arm parallel randomised trial (1:1 allocation ratio) conducted in collaboration with BMJ Open, an open-access journal that publishes a wide variety of medical research and requests the submission of completed CONSORT checklists for RCTs. Prior to recruitment, we registered the study in ClinicalTrials.gov with the identifier NCT03751878 and uploaded the study protocol (19).

For peer review only - http://bmjopen.bmj.com/site/about/guidelines.xhtml

Eligibility criteria

Manuscripts were eligible for inclusion if (i) they were original research articles reporting the results of an RCT submitted to BMJ Open, (ii) they had passed the first editorial filter and had been subsequently sent out for peer review, and (iii) authors of these manuscripts had provided a completed CONSORT checklist. Apart from the standard two-arm parallel RCTs, which are covered by the standard CONSORT guidelines (20), we also included RCTs that require the use of the official CONSORT extensions for different design aspects (cluster (21), non-inferiority and equivalence (22), pragmatic (23), N-of-1 trials (24), Pilot and feasibility (25), and within person trials (26)) and intervention types (herbal (27), non-pharmacologic (28), acupuncture (29) and Chinese herbal medicine formulas (30)) in all areas of clinical research. We excluded (i) studies that claimed to be RCTs but used deterministic allocation methods, and (ii) secondary trial analysis studies.

Interventions

The lead investigator (DB), a PhD student with background in statistics that had worked for two years on the topic of improving adherence to RGs, performed a three-step intervention. First, he assessed eight core items (see paragraph below) of the completed CONSORT checklist submitted by authors to see whether they were consistent with the information reported in the manuscript. Secondly, he produced a standardised report containing precise requests to be addressed by authors. This report (see example in Box 1) included a point by point description of the reporting inconsistencies found, requests to the authors to include the missing information, as well as examples extracted from the CONSORT Explanation and Elaboration document (20). Finally, DB uploaded the report to the manuscript management system of the journal (ScholarOne) to make it accessible to the manuscript handling editor, who included this additional report in the decision letter to authors alongside the standard peer review reports. Manuscripts randomised to the control group underwent the usual peer review process. In Figure 1, we display a schema of the study design.

For peer review only - http://bmjopen.bmj.com/site/about/guidelines.xhtml

This report shows the results of an evaluation of the consistency between the submitted CONSORT checklist and the information reported in the manuscript. The examples or cites included in the report were extracted from the CONSORT E&E Document (https://www.bmj.com/content/340/bmj.c869).

Please, make the following revisions:

- For CONSORT Item 8a ("*Method used to generate the random allocation sequence*"), please report the exact method you used to generate the random allocation sequence.
  - Example from CONSORT: "*Randomization sequence was created using Stata M.N (StataCorp, College Station, TX) statistical software*".
- For CONSORT Item 11a ("*If done, who was blinded after assignment to interventions and how*"), please specify in "Trial design and setting" who was blinded in the study and do not just state that it was a double-blind randomised trial.
  - Example from CONSORT: "*Whereas patients and physicians allocated to the intervention group were aware of the allocated arm, outcome assessors and data analysts were kept blinded to the allocation*"

Box 1: Example of report on the reporting inconsistencies found

Our intervention focused on eight core CONSORT items which are essential for systematic reviewers to evaluate the risk of bias (31) and which are usually poorly reported (32). These items are:

- Five items in the methods section:
  - Item 6a ("*Completely defined pre-specified primary and secondary outcome measures, including how and when they were assessed*")
  - Item 8a ("*Method used to generate the random allocation sequence*")
  - Item 9 ("*Mechanism used to implement the random allocation sequence (such as sequentially numbered containers), describing any steps taken to conceal the sequence until interventions were assigned*")

For peer review only - http://bmjopen.bmj.com/site/about/guidelines.xhtml

    - Item 11a ("*If done, who was blinded after assignment to interventions (for example, participants, care providers, those assessing outcomes) and how*")
    - Item 11b ("*If relevant, description of the similarity of interventions*")
- Three items in the results section:
    - Item 13a ("*For each group, the numbers of participants who were randomly assigned, received intended treatment, and were analysed for the primary outcome*")
    - Item 13b ("*For each group, losses and exclusions after randomisation, together with reasons*")
    - Item 17a ("*For each primary and secondary outcome, results for each group, and the estimated effect size and its precision (such as 95% confidence interval)*")

We considered an item as adequately reported if all subparts of it were adequately reported, according to the CONSORT E&E document (20) and the corresponding E&E documents for the extensions considered. For example, for CONSORT item 6a ("*Completely defined pre-specified primary and secondary outcome measures, including how and when they were assessed*"), we required the following subparts to be adequately reported: A) identified and completely defined primary and secondary outcomes, B) analysis metric and methods of aggregation for each outcome, and C) time points for each outcome.

The items corresponding to CONSORT extensions were assessed in addition to the standard CONSORT items. For example, we expected authors of a cluster randomised trial evaluating a pharmacologic treatment to be using the standard CONSORT checklist for all eight items and the cluster extension for items 6a, 9, 13a, 13b, and 17a. In contrast, the items requested by the Pilot and Feasibility extension substituted the standard CONSORT items, as specified in its E&E document (25). Once the recruitment was ongoing, we decided to discard the extension for non-pharmacologic interventions as it was not being requested by the editors, nor sent by authors.

In Supplementary File 1 we present further details on the criteria we used to evaluate the reporting inconsistencies, including rules on how to deal with N/As and certain aspects of specific items.

For peer review only - http://bmjopen.bmj.com/site/about/guidelines.xhtml

Outcomes

- **Primary outcome**: Mean score for completeness of reporting, defined as the mean number of adequately reported items in the first revised manuscript (0 to 8 scale).
- **Secondary outcome**: Proportion of manuscripts where each item was adequately reported.

In the design phase of the study, we considered two potential scenarios that could lead to missing data on the study outcomes: (i) when editors rejected a manuscript after peer review, and (ii) when authors did not return the revised manuscript within the period requested by the handling editor after a “Minor revision” or “Major revision” editorial decision (14 and 28 days, respectively, plus, if necessary, the extra time that the editor considered appropriate). We report the methods used to handle missing data in the “Statistical methods” section.

Outcome evaluation was performed independently and in duplicate by two senior researchers (EC, JJK) who were blinded to manuscript allocation and had experience as authors and reviewers of RCTs. They also assessed outcomes at baseline. In case that a manuscript was rejected after the first round of peer review, assessors could only evaluate it at baseline. However, they were not aware of the fate of that manuscript until they finished that evaluation. More details about the outcome assessment process can be found in Supplementary file 2.

For each of the manuscripts in the intervention group, we also recorded the amount of time that took the lead investigator to perform the assessment of reporting inconsistencies and to produce the report.

Harms

We analysed whether our intervention caused the following unintended effects: (i) higher proportion of manuscript rejections after the first round of peer review, and (ii) delays in the submission of the revised manuscripts by authors.

Pilot work

For peer review only - http://bmjopen.bmj.com/site/about/guidelines.xhtml

To inform the sample size calculation, the lead investigator performed a pilot evaluation of 12 randomly selected RCTs published in BMJ Open between April 2018 and September 2018. The observed proportions of adequately reported items in these manuscripts were used to estimate the scores for completeness of reporting of the manuscripts in the control group (usual peer review).

Furthermore, outcome assessors (EC and JJK) practised the evaluation of completeness of reporting by assessing six of the 12 RCTs mentioned above.

Prior to recruitment of manuscripts, daily DB screened automated reports listing of original research submissions to BMJ Open on ScholarOne, including their ID, date of submission, title, abstract, and different parameters related to their peer review status. RCTs were identified for possible inclusion based on the title and abstract and then checked against our eligibility criteria until the desired sample size was achieved.

Power analysis

According to our pilot evaluation (see “Pilot work” section), the estimated probabilities that manuscripts in the control group adequately reported 0, 1, 2,…, and 8 items were 0, 0, 0, 0, 0, 0.17, 0.33, 0.33, and 0.17, respectively. For manuscripts in the intervention group to adequately report 7 or 8 items 50% of the time respectively, a sample size of 24 articles (12 per arm) was enough to detect such a difference between groups with 90% power (see Supplementary file 3: Script A).

Randomisation and blinding

Every we detected a manuscript meeting our eligibility criteria (according to the submissions report mentioned above), DB introduced its ID into an R Shiny application (33) created by a senior statistician (JAG) (see Supplementary file 3: Script B), which randomised the manuscript to the intervention or the control group (1:1 allocation ratio, blocks of 4). Manuscripts were stratified according to whether there was an applicable CONSORT extension for that study or not. To avoid allocation bias, each ID could only be introduced once.

For peer review only - http://bmjopen.bmj.com/site/about/guidelines.xhtml

Authors of included manuscripts were unaware that their manuscripts were part of an RCT and the study was conducted as part of the journal's quality improvement programme. Outcome assessors were blinded to allocation and to each other's evaluation.

Handling editors of the included manuscripts and the investigator performing the intervention (DB) were not blinded.

Statistical methods

We carried out statistical analysis using R version 3.6.0 (34).

For the primary outcome, we adjusted a linear regression model with the baseline percentage of consistency between the manuscript and the checklist as the only covariate. We calculated the 95% confidence interval using bootstrapping, a simple yet powerful non-parametric technique that consists in creating multiple resamples from a set of observations (35). This allows the calculation of the differences between the groups for each of the resamples, which are used to easily compute the 95% confidence interval (see Supplementary File 3: Script C).

We performed the main analysis of the primary outcome imputing missing data with a value of *1-b*, where *b* was the baseline score of the manuscript, regardless of the manuscript allocation. This aimed to reflect the decision not to publish an RCT that is not transparent and accurate enough to be considered an editorial success. Secondarily, we assessed the robustness of the main analysis results by carrying out the complete case analysis.

Deviations from the protocol

The last inclusion criteria mentioned above ((iii) authors of the manuscripts had provided a completed CONSORT checklist) was included before recruitment started. The reason was that, despite that the submission of CONSORT checklist for trials is mandatory, we observed that handling editors were occasionally overlooking this requirement and sending out manuscripts of trials for peer review that did not include one. Second, we initially used a t-test to calculate the study power and planned to use it for the primary outcome analysis. However, to relax the strong assumptions behind it for a relatively small sample size, we re-defined the main analysis to be performed using bootstrapping (see "Statistical methods" section) and checked that the power did not decrease. Furthermore, we decided to collect

For peer review only - http://bmjopen.bmj.com/site/about/guidelines.xhtml

baseline scores for completeness of reporting for the included manuscripts in order to adjust for these in the primary outcome analysis.

Reporting guidelines

We report this manuscript in accordance to CONSORT 2010 (6).

Patient and public involvement

No patients or public were involved in the study.

Results

Between 31 October 2018 and 4 April 2019, we screened 62 manuscripts that described RCTs submitted to BMJ Open. Among these, we excluded 38 either because they were rejected without peer review (n = 34) or because the authors did not provide the CONSORT checklist (n = 4). We randomised the remaining 24 to the intervention (n = 12) or control (n = 12) groups. Figure 2 shows the flow diagram of the study.

We had missing data on the primary outcome for six (25%) of the manuscripts (intervention n = 3, control n = 3). These were rejected after the first round of peer review and therefore not returned to authors with a request for revision (type (i) of missing data, see "Outcomes" section). We had no missing data of type (ii) as all authors returned the revised manuscripts within the established time. While only 18 manuscripts were revised by authors, we included all 24 manuscripts in the main analysis.

Most (19, 70%) of the 24 included manuscripts required the use of at least one of the extensions considered: non-pharmacologic (intervention n = 9; control n = 8), pilot and feasibility (n = 3; n = 4), cluster (n = 2; n = 2).

The mean (SD) baseline score for completeness of reporting (0 to 8 scale) prior to peer review in the intervention and control groups (n = 24) was 4.35 (1.88) and 4.85 (1.79), respectively. The mean (SD) baseline score of the manuscripts that later passed the first round of peer review (n = 18) almost doubled the score of the ones that were rejected after the first round of peer review (n = 6): 5.23 (1.35) versus 2.68 (1.75).

For peer review only - http://bmjopen.bmj.com/site/about/guidelines.xhtml

Primary outcome

The manuscripts that received the intervention were more completely reported than the ones that underwent the standard review process: intervention group (mean: 7.01; SD: 1.47) versus control group (mean: 5.68; SD: 1.43). After adjusting for the baseline score, the mean difference in scores between the two groups (n = 24) was 1.43 (95% CI: 0.31 to 2.58). These results imply that the manuscripts in the intervention group reported in average 1.43 (out of 8) items more than those receiving the standard peer review. For the complete case (n = 18), the mean (SD) scores for the intervention and control groups were 7.45 (1.00) and 5.90 (1.35), yielding an adjusted difference of 1.75 (95% CI: 0.80 to 2.75). Table 1 summarises these results.

Figure 3 shows the evolution of the 18 manuscripts that passed the first round of peer review. From the nine manuscripts in the intervention group, six of them achieved the maximum score and the other two improved. In contrast, the only manuscript in the control group that reached the maximum score already had that score at baseline. Three manuscripts in the control group slightly improved (1, 1, and 2 items respectively). We identified that 3 out of 4 of these improvements came from comments that were made by the standard peer reviewers.

Secondary outcome

Figure 4 displays the proportions of manuscripts where each CONSORT item was adequately reported. We observed the main differences favouring the intervention group in items 6a (Outcomes), 9 (Allocation concealment mechanism), 11a (Blinding), and 17a (Outcomes and estimation).

The mean (SD) time taken to evaluate the consistency between the submitted checklist and the manuscript, and to produce the report was 87 (42) minutes. There was no correlation between the amount of time taken and the baseline score of the manuscript (ρ = 0.08).

Harms

We did not identify any unintended effects. There were not differences between the intervention and the control groups for (i) the proportion of manuscripts that were rejected

For peer review only - http://bmjopen.bmj.com/site/about/guidelines.xhtml

after the first round of peer review (3 of 12, 25%), which was similar to the historical rates of the journal, and (ii) the timeliness of submitting their revisions, as all authors submitted the revised manuscripts within the period requested by the handling editor.

Discussion

This study found that the introduction of an editorial intervention performed by a CONSORT expert during peer review significantly improved the completeness of reporting of trials submitted to BMJ Open, compared to the standard peer review process. As a result of the intervention, an average (95% CI) of 1.43 (0.31 to 2.58) more items (out of 8) were reported in the intervention group. Moreover, providing authors with extra comments on reporting issues did not seem to discourage them from revising the manuscript as all authors returned the revised manuscripts within the standard 28 days.

Strengths and limitations

This study has several strengths: the randomised trial design; the fact that the intervention was performed in a real editorial context with no disruption to normal editorial procedures; and the fact that the outcome assessment process was blinded and in duplicate.

We also note some limitations. First, our intervention was focused only on one RG. As other RGs are less established and worse understood by authors than CONSORT, it could potentially be more difficult for them to properly address reviewers' comments. Second, we only considered one journal and the same effect might not be observed in other journals. However, we purposefully selected a very large general journal receiving international submissions across multiple specialties. Furthermore, we considered only eight core CONSORT items and not the whole checklist. However, the ones we chose are known to be usually poorly reported (32).

Implications

Given the importance of improving the completeness of reporting of randomised trials and given the ineffectiveness of the strategies that biomedical journals are currently implementing, it is time to take a step forward. Our study provides empirical evidence of the fact that involving a CONSORT expert in the peer review process has a major impact on the

For peer review only - http://bmjopen.bmj.com/site/about/guidelines.xhtml

completeness of randomised trials. In this case, the role of reporting expert was played by a PhD student with expertise on CONSORT who acted as an additional peer reviewer. Also, this could be done by editorial assistants, administrators, text editors or external consultants.

More than two decades ago, scientists started to discuss the importance of including statistical reviews as part of the publication process (36). Nowadays, statistical reviews have become widespread among top medical journals. These are usually performed by a statistician and focus on the methodological and statistical aspects of the study. As methodological issues are often not fixable, statistical reviews are key to determine the fate of manuscripts as they help prevent unsound research getting published (37). Completeness of reporting reviews should also be a key component in the publication system. As reporting issues are usually improvable, these reviews should not generally aim to determine whether a manuscript should be published or not, but to improve their transparency. This would both help editors and peer reviewers make decisions on the manuscripts and improve the usability of published papers.

The time taken for us to perform the intervention (87 minutes on average, with great variations from manuscript to manuscript) is a barrier to wider implementation. However, the great benefits it provided should encourage journal editors to think about how they might put it into practice and tailor it to their preferences. For example, the intervention could be focused only on RGs whose implementation is proven to improve the completeness of reporting. While the results for CONSORT in this study are conclusive, it is still to be proven whether similar benefits could be obtained in the case of other popular RGs, such as SPIRIT (38) or PRISMA (39). Second, this intervention could be performed at other points in the editorial process: (i) before the first decision on the manuscript, or (ii) between the first decision and the start of the peer review process. For this study, we discarded both options for pragmatic reasons, as we did not want to alter the normal editorial process. While the first could be too resource intensive for journals, the latter would imply the same effort and the manuscript would undergo peer review being more transparent and accurate, which could make the task of peer reviewers and handling editors easier and more efficient.

Conclusions

For peer review only - http://bmjopen.bmj.com/site/about/guidelines.xhtml

This study provides evidence that engaging experts in the process of evaluating RG checklists submitted by authors has a great impact on the completeness of reporting of randomised trials. This is essential to reduce research waste associated to the inadequate reporting of RCT methods and findings. Journal editors should consider revising their peer review processes to find ways to make this intervention workable, tailoring it to their preferences.

Acknowledgements

We thank the MiRoR Project and Marie Sklodowska-Curie Actions for their support. This RCT is the third part of a larger project whose first part was a scoping review to identify and classify interventions to improve adherence to RGs (8) and whose second part was a survey to explore biomedical editors' opinion on various editorial interventions to improve adherence to RGs (18).

We thank BMJ Open for collaborating with this project, and also José Antonio González and Jordi Cortés (Universitat Politècnica de Catalunya) for collaborating in the process of developing the R codes used to perform the randomisation and outcome analysis.

Funding and role of funders

This study is part of the ESR 14 research project from the Methods in Research on Research (MiRoR) project (http://miror-ejd.eu/), which has received funding from the European Union's Horizon 2020 research and innovation programme under the Marie Sklodowska-Curie grant agreement No 676207. DM is supported through a University Research Chair (University of Ottawa). The sponsor and the funding source had no role in the design of this study and will not have any role during its execution, analyses, interpretation of the data, or decision to submit results.

Contributor roles

Contributor roles are reported according to the Contributor Roles Taxonomy (CRediT) (40).

DB: Conceptualisation, Methodology, Software, formal analysis, Investigation, writing – original draft preparation

SS: Conceptualisation, Methodology, Resources, Writing – review and editing

AA: Conceptualisation, Methodology, Resources, Writing – review and editing

DM: Conceptualisation, Methodology, Writing – review and editing

For peer review only - http://bmjopen.bmj.com/site/about/guidelines.xhtml

IB: Conceptualisation, Methodology, Writing – review and editing

JJK: Conceptualisation, Methodology, Outcome evaluation; Writing – review and editing, Supervision

EC: Conceptualisation, Methodology, Outcome evaluation; Writing – review and editing, Supervision

Ethics approval and informed consent

We obtained ethics approval from the Research Committee of the Governing Council of the Universitat Politècnica de Catalunya (UPC). Ref: EC 02. Date: 13 September 2018.

All authors of the submitted manuscripts were informed that BMJ has a research programme and that they could opt out if they wished. Moreover, all authors and reviewers are told to read the BMJ Company Privacy Statement, which describes the fact that BMJ Publishing Group has a research programme of quality improvement of peer review.

Data management and confidentiality

All data related to the study were collated and managed from a password protected spreadsheet file stored in a BMJ Google Drive folder.

For DB to access BMJ Open's manuscript tracking system, a confidentiality agreement with BMJ Publishing Group was signed to certify that (i) BMJ Publishing group wished to disclose information to DB, and (ii) DB wished to receive this information on a confidential basis.

Data sharing

The content of the intervention reports reflecting reporting inconsistencies will appear as part of the peer review history of the manuscripts included in the study. However, in order to protect confidentiality, we are not releasing any dataset including individual manuscript data or outcome data identifying the performance of individual participants.

Declaration of interests

AA is Editor in Chief of BMJ Open. SS is Senior Researcher at The BMJ. DM is Director of the Canadian EQUATOR Centre. IB is deputy director of French EQUATOR Centre. DM, IB, and EC are members of the CONSORT steering group.

Transparency statement

For peer review only - http://bmjopen.bmj.com/site/about/guidelines.xhtml

For peer review only

The lead author affirms that this manuscript is an honest, accurate, and transparent account of the study being reported; that no important aspects of the study have been omitted; and that any discrepancies from the study as planned (and, if relevant, registered) have been explained.

Figures and tables

Figure 1: Schema of the study design.

Figure 2: CONSORT flow diagram.

Figure 3: Evolution of the scores for each manuscript.

Figure 4: Proportion of manuscripts where each CONSORT item is adequately reported. **Legend**: Cont: control group; Int: intervention group. CONSORT items:

- 6a ("*Completely defined pre-specified primary and secondary outcome measures, including how and when they were assessed*")
- 8a ("*Method used to generate the random allocation sequence*")
- 9 ("*Mechanism used to implement the random allocation sequence (such as sequentially numbered containers), describing any steps taken to conceal the sequence until interventions were assigned*")
- 11a ("*If done, who was blinded after assignment to interventions (for example, participants, care providers, those assessing outcomes) and how*")
- 11b ("*If relevant, description of the similarity of interventions*")
- 13a ("*For each group, the numbers of participants who were randomly assigned, received intended treatment, and were analysed for the primary outcome*")
- 13b ("*For each group, losses and exclusions after randomisation, together with reasons*")
- 17a ("*For each primary and secondary outcome, results for each group, and the estimated effect size and its precision (such as 95% confidence interval)*")

For peer review only - http://bmjopen.bmj.com/site/about/guidelines.xhtml

Table 1: Scores for completeness of reporting scores in the control and intervention group.

Outcome	Intervention<br>Mean (SD)	Control<br>Mean (SD)	Mean difference*<br>(95% CI)
Completeness of reporting (0 to 8 scale) with imputation (n = 24)	7.01 (1.47)	5.68 (1.79)	1.43 (0.31 to 2.58)
Completeness of reporting (0 to 8 scale) without imputation (complete case analysis, n = 18)	7.45 (1.00)	5.90 (1.35)	1.75 (0.80 to 2.75)

*Adjusted for baseline score.

Supplementary files

Supplementary file 1: Rules for the evaluation of reporting inconsistencies.

Supplementary file 2: Further details of the outcome assessment process.

Supplementary file 3: R scripts.

For peer review only - http://bmjopen.bmj.com/site/about/guidelines.xhtml

References

1. Glasziou P, Altman DG, Bossuyt P, Boutron I, Clarke M, Julious S, et al. Reducing waste from incomplete or unusable reports of biomedical research. Lancet. 2014 Jan 18;383(9913):267–76.

2. EQUATOR Network. What is a reporting guideline? [Internet]. 2019 [cited 2019 Nov 28]. Available from: http://www.equator-network.org/about-us/what-is-a-reporting-guideline/

3. Begg C, Cho M, Eastwood S, Horton R, Moher D, Olkin I, et al. Improving the quality of reporting of randomized controlled trials. The CONSORT statement. JAMA. 1996 Aug 28;276(8):637–9.

4. EQUATOR Network. Library for health research reporting [Internet]. 2019 [cited 2019 Dec 2]. Available from: https://www.equator-network.org/library/

5. Altman DG, Schulz KF, Moher D, Egger M, Davidoff F, Elbourne D, et al. The revised CONSORT statement for reporting randomized trials: Explanation and elaboration. Ann Intern Med. 2001 Apr 17;134(8):663–94.

6. Schulz KF, Altman DG, Moher D, CONSORT Group. CONSORT 2010 statement: updated guidelines for reporting parallel group randomised trials. BMJ. 2010 Mar 23;340:c332.

7. Samaan Z, Mbuagbaw L, Kosa D, Borg Debono V, Dillenburg R, Zhang S, et al. A systematic scoping review of adherence to reporting guidelines in health care literature. J Multidiscip Healthc. 2013 May;6:169–88.

8. Blanco D, Altman D, Moher D, Boutron I, Kirkham JJ, Cobo E. Scoping review on interventions to improve adherence to reporting guidelines in health research. BMJ Open. 2019;9(5).

9. Cobo E, Selva-O'Callagham A, Ribera JM, Cardellach F, Dominguez R, Vilardell M. Statistical reviewers improve reporting in biomedical articles: A randomized trial. PLoS One. 2007;2(3):e332.

10. Cobo E, Cortés J, Ribera JM, Cardellach F, Selva-O'Callaghan A, Kostov B, et al. Effect of using reporting guidelines during peer review on quality of final manuscripts submitted to a biomedical journal: masked randomised trial. BMJ. 2011 Nov 22;343(nov22 2):1084.

11. Barnes C, Boutron I, Giraudeau B, Porcher R, Altman DG, Ravaud P. Impact of an online

For peer review only - http://bmjopen.bmj.com/site/about/guidelines.xhtml

writing aid tool for writing a randomized trial report: the COBWEB (Consort-based WEB tool) randomized controlled trial. BMC Med. 2015 Sep 15;13(1):221.

12. Hopewell S, Boutron I, Altman DG, Barbour G, Moher D, Montori V, et al. Impact of a web-based tool (WebCONSORT) to improve the reporting of randomised trials: results of a randomised controlled trial. BMC Med. 2016 Nov 28;14(1):199.

13. Turner L, Shamseer L, Altman DG, Schulz KF, Moher D. Does use of the CONSORT Statement impact the completeness of reporting of randomised controlled trials published in medical journals? A Cochrane review. Syst Rev. 2012 Nov 29;1(1):60.

14. Stevens A, Shamseer L, Weinstein E, Yazdi F, Turner L, Thielman J, et al. Relation of completeness of reporting of health research to journals' endorsement of reporting guidelines: systematic review. BMJ. 2014 Jun 25;348(jun25 2):g3804–g3804.

15. Blanco D, Biggane AM, Cobo E. Are CONSORT checklists submitted by authors adequately reflecting what information is actually reported in published papers? Trials. 2018 Dec 29;19(1):80.

16. Hair K, Macleod MR, Sena ES. A randomised controlled trial of an Intervention to Improve Compliance with the ARRIVE guidelines (IICARus). Res Integr Peer Rev. 2019 Dec;4(1).

17. Pandis N, Shamseer L, Kokich VG, Fleming PS, Moher D. Active implementation strategy of CONSORT adherence by a dental specialty journal improved randomized clinical trial reporting. J Clin Epidemiol. 2014 Sep;67(9):1044–8.

18. Blanco D, Hren D, Kirkham JJ, Cobo E, Schroter S. A survey exploring biomedical editors' perceptions of editorial interventions to improve adherence to reporting guidelines. F1000Research. 2019 Sep 24;8:1682.

19. Blanco D, Schroter S, Moher D, Aldcroft A, Boutron I, Kirkham JJ, et al. Evaluating the Impact of Assessing During Peer Review the CONSORT Checklist Submitted by Authors [Internet]. 2019 [cited 2019 Nov 28]. Available from: https://clinicaltrials.gov/ct2/show/NCT03751878

20. Moher D, Hopewell S, Schulz KF, Montori V, Gøtzsche PC, Devereaux PJ, et al. CONSORT 2010 explanation and elaboration: updated guidelines for reporting parallel group randomised trials. BMJ. 2010;340.

21. Campbell MK, Piaggio G, Elbourne DR, Altman DG, CONSORT Group. Consort 2010 statement:

For peer review only - http://bmjopen.bmj.com/site/about/guidelines.xhtml

extension to cluster randomised trials. BMJ. 2012 Sep 4;345:e5661.

22. Piaggio G, Elbourne DR, Pocock SJ, Evans SJW, Altman DG. Reporting of noninferiority and equivalence randomized trials: Extension of the CONSORT 2010 statement. Vol. 308, JAMA - Journal of the American Medical Association. 2012. p. 2594–604.

23. Zwarenstein M, Treweek S, Gagnier JJ, Altman DG, Tunis S, Haynes B, et al. Improving the reporting of pragmatic trials: an extension of the CONSORT statement. BMJ. 2008;337(nov11 2):a2390.

24. Vohra S, Shamseer L, Sampson M, Bukutu C, Schmid CH, Tate R, et al. CONSORT extension for reporting N-of-1 trials (CENT) 2015 Statement. J Clin Epidemiol. 2016;76:9–17.

25. Eldridge SM, Chan CL, Campbell MJM, Bond CM, Hopewell S, Thabane L, et al. CONSORT 2010 statement: Extension to randomised pilot and feasibility trials. BMJ. 2016;355.

26. Pandis N, Chung B, Scherer RW, Elbourne D, Altman DG. CONSORT 2010 statement: Extension checklist for reporting within person randomised trials. BMJ. 2017;357.

27. Gagnier JJ, Boon H, Rochon P, Moher D, Barnes J, Bombardier C. Reporting Randomized, Controlled Trials of Herbal Interventions: An Elaborated CONSORT Statement. Ann Intern Med. 2013 Mar 7;144(5):364.

28. Boutron I, Moher D, Altman DG, Schulz KF, Ravaud P. Extending the CONSORT Statement to Randomized Trials of Nonpharmacologic Treatment : Explanation and Elaboration. Ann Intern Med. 2008;148(4):295–309.

29. MacPherson H, Altman DG, Hammerschlag R, Youping L, Taixiang W, White A, et al. Revised STandards for Reporting Interventions in Clinical Trials of Acupuncture (STRICTA): Extending the CONSORT Statement. PLoS Med. 2010 Jun 8;7(6):e1000261.

30. Cheng C, Wu T, Shang H, Li Y, Altman DG, Moher D, et al. CONSORT Extension for Chinese Herbal Medicine Formulas 2017: Recommendations, Explanation, and Elaboration. Ann Intern Med. 2018;167(2):112.

31. Chauvin A, Moher D, Altman D, Schriger DL, Alam S, Hopewell S, et al. A protocol of a cross-sectional study evaluating an online tool for early career peer reviewers assessing reports of randomised controlled trials. BMJ Open. 2017 Sep 15;7(9):e017462.

For peer review only - http://bmjopen.bmj.com/site/about/guidelines.xhtml

32. Hopewell S, Collins GS, Boutron I, Yu L-M, Cook J, Shanyinde M, et al. Impact of peer review on reports of randomised trials published in open peer review journals: retrospective before and after study. BMJ. 2014;349.

33. González J-A. Randomisation of BMJ Open manuscripts [Internet]. 2019 [cited 2019 Dec 29]. Available from: http://shiny-eio.upc.edu/MIRoR/randomassign/

34. R Core Team. R: A language and environment for statistical computing [Internet]. 2019. Available from: http://www.r-project.org/

35. Efron B. Bootstrap Methods: Another Look at the Jackknife. Ann Stat. 1979 Jan;7(1):1–26.

36. Altman DG. Statistical reviewing for medical journals. Vol. 17, Ltd. Statistics in Medicine Statist. Med. John Wiley & Sons; 1998.

37. Petrovečki M. The role of statistical reviewer in biomedical scientific journal. Biochem Medica. 2009;19(3).

38. Chan A-W, Tetzlaff JM, Altman DG, Laupacis A, Gøtzsche PC, Krleža-Jerić K, et al. SPIRIT 2013 statement: Defining standard protocol items for clinical trials. Ann Intern Med. 2013;158(3):200–7.

39. Moher D, Liberati A, Tetzlaff J, Altman DG. Preferred reporting items for systematic reviews and meta-analyses: The PRISMA statement. Vol. 339, BMJ (Online). 2009. p. 332–6.

40. CASRAI. CRediT – Contributor Roles Taxonomy [Internet]. [cited 2019 Dec 29]. Available from: https://casrai.org/credit/

For peer review only

For peer review only - http://bmjopen.bmj.com/site/about/guidelines.xhtml

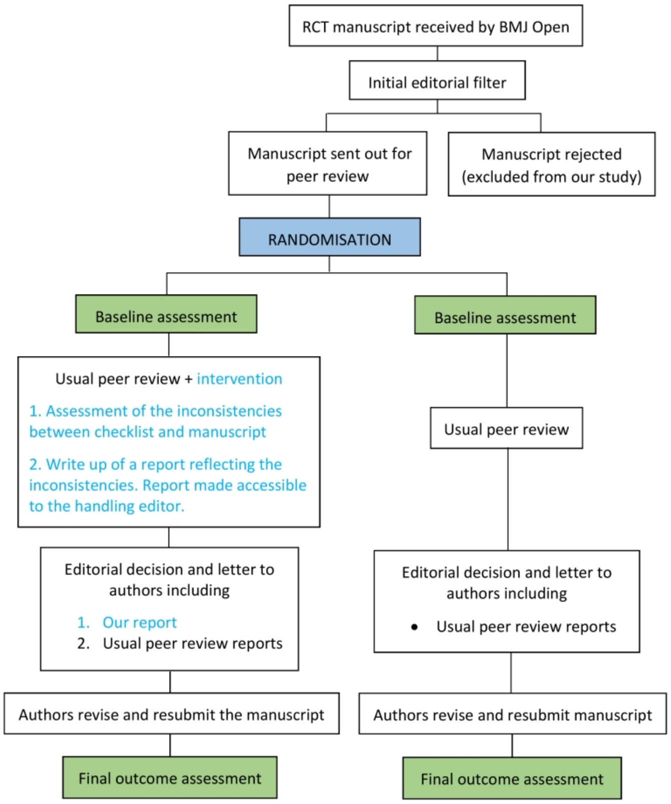

Schema of the study design

209x297mm (300 x 300 DPI)

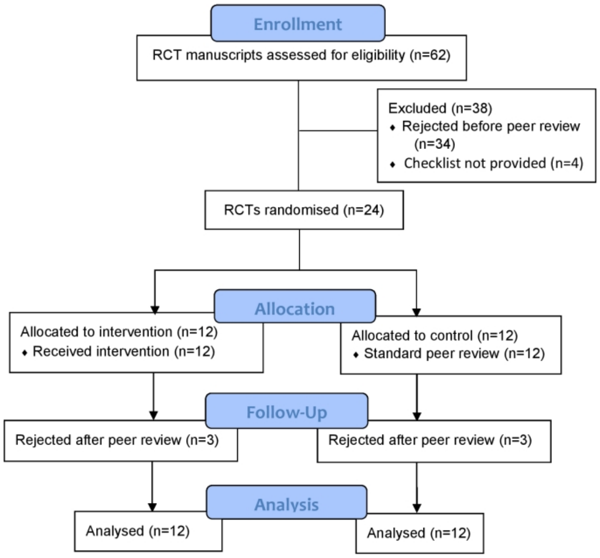

CONSORT flow diagram

209x297mm (300 x 300 DPI)

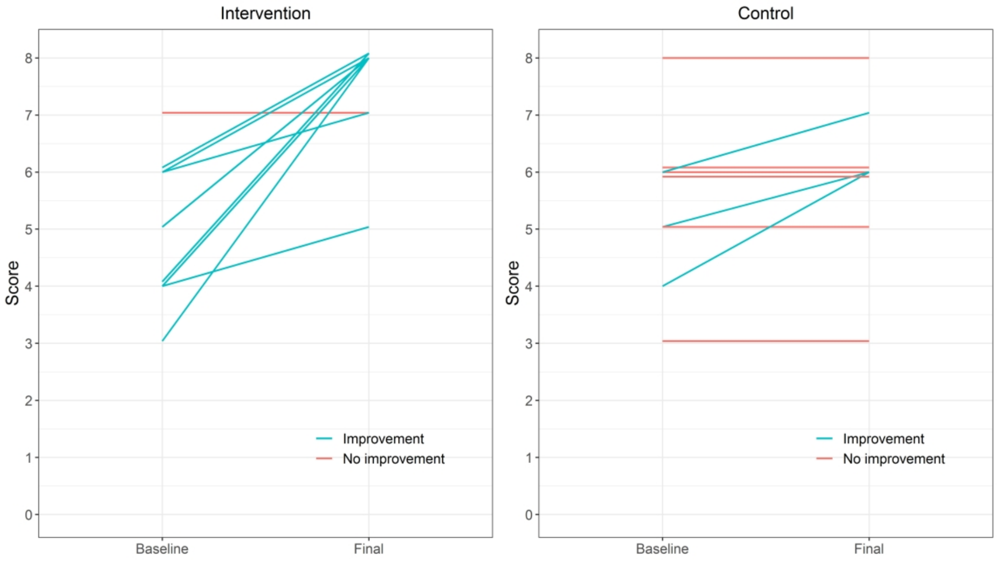

Evolution of the scores for each manuscript

304x177mm (300 x 300 DPI)

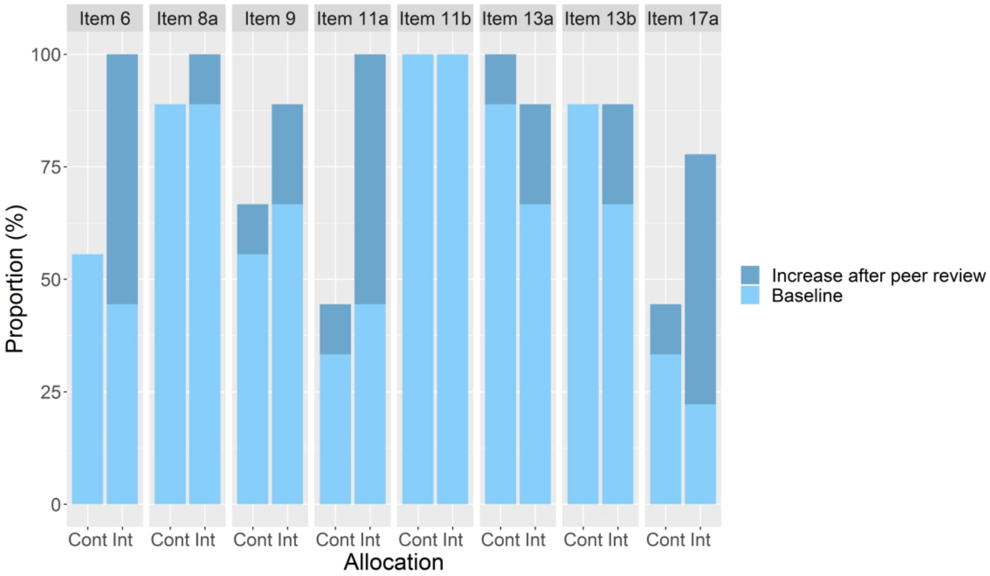

Proportion of manuscripts where each CONSORT item is adequately reported. Legend: Cont: control group; Int: intervention group. CONSORT items:

• 6a ("Completely defined pre-specified primary and secondary outcome measures, including how and when they were assessed")

• 8a ("Method used to generate the random allocation sequence")

• 9 ("Mechanism used to implement the random allocation sequence (such as sequentially numbered containers), describing any steps taken to conceal the sequence until interventions were assigned")

• 11a ("If done, who was blinded after assignment to interventions (for example, participants, care providers, those assessing outcomes) and how")

• 11b ("If relevant, description of the similarity of interventions")

• 13a ("For each group, the numbers of participants who were randomly assigned, received intended treatment, and were analysed for the primary outcome")

• 13b ("For each group, losses and exclusions after randomisation, together with reasons")

• 17a ("For each primary and secondary outcome, results for each group, and the estimated effect size and its precision (such as 95% confidence interval)")

304x177mm (300 x 300 DPI)

N/As for a certain item

For items reported as N/A in the CONSORT checklist, we consider them as:

- Adequately reported if (i) the item did not apply and therefore it did not have to be reported, and (ii) the item applied and it was actually reported although the page number was not given.
- Inadequately reported if the item did apply but it was not adequately reported.

Rules about specific items:

- **Item 8a** (“*Method used to generate the random allocation sequence*”): inadequately reported if authors have reported this information elsewhere but not in the main body of the article. According to CONSORT, “*it is important that information on the process of randomisation is included in the body of the main article and not as a separate supplementary file; where it can be missed by the reader*”.
- **Item 11a** (“*If done, who was blinded after assignment to interventions (for example, participants, care providers, those assessing outcomes) and how*”): adequately reported if blinding was not performed and authors explicitly said so, and inadequately reported if blinding was assumed to be not performed and authors did not mention it in the manuscript.
- **Item 13a** (“*For each group, the numbers of participants who were randomly assigned, received intended treatment and were analysed for the primary outcome*”) and **item 13b** (“*For each group, losses and exclusions after randomisation, together with reasons*”)**:** the corresponding information could be included either in the text or in the flow diagram. If information was only included in the discussion, it was considered as inadequately reported.
- **Item 17a** (“*For each primary and secondary outcome, results for each group, and the estimated effect size and its precision (such as 95% confidence interval)*”)**:** adequately reported if there was a correspondence between the outcomes in the results section and the ones listed in the methods section (and therefore evaluated in Item 6a).
- **Extension of Item 17a** for Pilot and Feasibility trials (“*For each objective, results including expressions of uncertainty (such as 95% confidence interval) for any estimates. If relevant, these results should be by randomised group*”): we did not expect authors to

For peer review only

report the effect sizes but the results (plus expressions of uncertainty) for each objective.

For peer review only

We divided the 24 included manuscripts into 4 batches of 6 manuscripts.

Every time DB detected in the submissions report (see “Preliminary work” section) that all 6 manuscripts of each batch had been revised by authors, he first made available to the outcome assessors the submitted version of the manuscript (version 1). Assessors had to complete the evaluation form for each manuscript independently and in duplicate. This form included the CONSORT extensions to be used. Assessors could explicitly indicate in it that they wanted to discuss a specific item with the other assessor. Once they were done with all manuscripts’ version 1, DB informed them of the discrepancies between their evaluations, which were resolved by consensus. Afterwards, he shared the manuscript revised by the authors (version 2) and we repeated the outcome evaluation process.

This process was done for the 4 batches of 6 manuscripts.

For peer review only

Script A: Power calculation

```
na <- 12
nb <- 12
A <- 0:8
B <- 0:8
#Estimated probabilities of reporting 0,...,8 items in the intervention group (a) and control group (b)
pra <- c(0, 0, 0, 0, 0, 0, 0, 5, 5)
pra <- pra/sum(pra)
prb <- c(0, 0, 0, 0, 0, 2, 4, 4, 2)
prb <- prb/sum(prb)
nboot <- 1000
N <- 1000
#Matrices containing random samples of scores for the two groups
ma <- matrix(sample(A, pr = pra, replace = TRUE, siz = N * na), ncol = N)
mb <- matrix(sample(B, pr = prb, replace = TRUE, siz = N * nb), ncol = N)
#Generation of N confidence intervals
sign1 <- c()
for (i in 1:N) {
 reporting <- data.frame(
  score = c(ma[, i], mb[, i]),
  group = factor(rep(c("Control", "Intervention"), c(na, nb)))
 )
 # Bootstrapping
 diff.mean1 <- c()
 for (k in 1:nboot){
  sel <- sample(1:(na + nb), na + nb, rep = TRUE)  # selected articles
  reporting.boot <- reporting[sel, ]
  diff.mean1[k]<- with(reporting.boot, diff(tapply(score, group, mean)))
 }
 conf.int1 <- quantile(diff.mean1, c(0.025, 0.975))
 sign1[i] <- conf.int1[2] < 0 #Checking if the CI crosses 0
}
power <- sum(sign1)/N
```

Script B: Randomisation of manuscripts (R Shiny application)

```
#File 1
Sys.setlocale("LC_ALL", "es_ES.UTF-8") #to be sure that accents in text will be allowed in plots
library(shiny)
library(shinyalert)
fluidPage(
  useShinyalert(),
  fluidRow(
        headerPanel('Randomisation of BMJ Open manuscripts'),
        wellPanel(
                textInput("identif", "Please enter the manuscript ID:", width='33%'),
                textAreaInput("titulo", "Please enter the manuscript title, or at least its first words:"),
                selectInput("tipo", "Does it correspond to an extension of CONSORT?", choices=c("", "Yes",
"No"), width='33%'),
                actionButton("send", "SUBMIT")
        ),
        p() ,
        wellPanel(
                #h3('Data'),
                #p("(just for testing purpose)"),
                #tableOutput("asig"),
                #actionButton("borrar", "RESET")
        )
  )
```

```
#File 2
library(shiny)
library(shinyalert)
library(blockrand)
is.void = function(x) {
   if (is.null(x)) return(TRUE)
         x == ''
}
shinyServer(function(input, output, session) {
upda = reactiveValues(asg=NA)
archi = "asignacion.dat"
```

```
#File 2 (continuation)

if (file.exists(archi)) {
        upda$asg = read.table(archi, header=TRUE, sep='\t', stringsAsFactors=FALSE)
} else {
   upda$asg = data.frame()
}
set.seed(6374422)
ext <- blockrand(n=100, id.prefix='E', block.prefix='B',stratum='Extension', block.sizes=c(2,2))
noext <- blockrand(n=100, id.prefix='N', block.prefix='B',stratum='No Ext.', block.sizes=c(2,2))
  go <- eventReactive(input$send, {
    list(id=input$identif, tit=input$titulo, ex=input$tipo)
  })
block_random = function(tip) {
  if (dim(upda$asg)[1]>0) {
     X = subset(upda$asg, strat==tip)
         x = dim(X)[1]
  } else x = 0
  if (tip=="Yes") {
                g = ext$treatment[x+1]
        } else if (tip=="No") {
                g = noext$treatment[x+1]
        } else return(-1)    # Error
  ifelse(g=='A', 0, 1)
}
        observeEvent(input$send, {
                Q = go()
                if (is.null(Q) | is.void(Q)) {
                        shinyalert(title="Please fill in the input.", type="error", showConfirmButton=TRUE, confirmButtonText="OK", timer=0)
                        return()
                }
                id = Q$id
                check.id = grep("^bmjopen-201[89]-[0-9]{6}$", id)
                if (length(check.id) == 0) {
                        shinyalert(title="Wrong ID.", text="Please enter a valid BMJ code.", type="error", showConfirmButton=TRUE, confirmButtonText="OK", timer=0)
return()
                }
```

```
#File 2 (continuation)

id = Q$id

        check.id = grep("^bmjopen-201[89]-[0-9]{6}$", id)

        if (length(check.id) == 0) {

            shinyalert(title="Wrong ID.", text="Please enter a valid BMJ code.", type="error", showConfirmButton=TRUE, confirmButtonText="OK", timer=0)

            return()

        }

        if (Q$ex=='') {

            shinyalert(title="Empty field:", text="extension of CONSORT?", type="error", showConfirmButton=TRUE, confirmButtonText="OK", timer=0)

            return()

        }

        if (Q$tit=='') {

            shinyalert(title="Empty field:", text="please provide a title.", type="error", showConfirmButton=TRUE, confirmButtonText="OK", timer=0)

            return()

        }

        n = dim(upda$asg)[1]

        if (n>0) {

            I = which(upda$asg$id==id)

            if (length(I)>0) {

                shinyalert(title="Invalid ID:", text="this ID has been already assigned.", type="error", showConfirmButton=TRUE, confirmButtonText="OK", timer=0)

                return()

            }

        }

        txt = paste("Go on with the manuscript '<i>", Q$tit, "</i>', with ID <b>",Q$id, "</b>, which <b>", ifelse(Q$ex=='Yes', 'corresponds', 'does not correspond'), "</b> to an extension of CONSORT:", sep='')

        shinyalert(title='Confirm inclusion', text=txt, closeOnEsc=TRUE, closeOnClickOutside=FALSE, html=TRUE, type="warning", showConfirmButton=TRUE, showCancelButton=TRUE, confirmButtonText="Right, go on", cancelButtonText="NO, stop", timer=0, imageUrl="", callbackR = Success)

    })

    Success = function(x) if (x != FALSE) {

        Q = go()

        g = block_random(Q$ex)

        if (g == -1) return

        L = list(id=Q$id, title=Q$tit, strat=Q$ex, group=g, date=date())

        upda$asg = rbind(upda$asg, as.data.frame(L))

        write.table(upda$asg, archi, sep='\t', row.names=FALSE)
```

```
#File 2 (continuation)

			filename = tempfile()

			interv = ifelse(g==0, "CONTROL (0)", "INTERVENTION (1)")

			cat(sprintf("Manuscript ID: %s\nTitle: %s\nExtension of CONSORT: %s\nAssigned to: %s\n",

				Q$id, Q$tit, Q$ex, interv), file=filename)

			# preparar y mandar mensaje

			dest = 'david.blanco@hotmail.com'

			Msg = tempfile()

			comm = paste('echo "To:', dest, '\nFrom: jose.a.gonzalez@upc.edu\nSubject: A manuscript has been assigned\n"| (cat -', filename, ') >', Msg)

			system(comm)

			system(paste("ssmtp", dest, "<", Msg))



			updateTextInput(session, "identif", "Please enter the manuscript ID:", value='')

			updateTextAreaInput(session, "titulo", "Please enter the manuscript title, or at least its first words:", value='')

			updateSelectInput(session, "tipo", "Does it correspond to an extension of CONSORT?", choices=c("", "Yes", "No"))

		}



		observeEvent(input$borrar, {

			shinyalert(title='Are you sure?', text="This action will remove the assignations.", closeOnEsc=TRUE, closeOnClickOutside=FALSE, html=TRUE, type="warning", showConfirmButton=TRUE, showCancelButton=TRUE, confirmButtonText="Yes, remove them", cancelButtonText="NO, don't reset", timer=0, imageUrl="", animation=TRUE, callbackR = function(x) { if(x != FALSE) { upda$asg = data.frame(); file.remove(archi) } })

		})

		output$asig = renderTable(  {

			upda$asg

		} )

})
```

Script C: Primary outcome analysis

```
# Loading the data

data <- read.csv2('Scores.txt', header = TRUE, sep = '')

data2 <- subset(data, data$Imputation==0)

# Fitting a linear model and calculating CIs with imputation

model1 <- lm(data$Final ~ data$Baseline + data$Group)

summary(model1)

na <- 12

nb <- 12

nboot <- 10000

set.seed(111111)

diff.mean1 <- c()

for (k in 1:nboot){

  sel <- sample(1:(na + nb), na + nb, rep=TRUE)  # selected articles

  reporting.boot <- data[sel, ]

  diff.mean1[k] <- coefficients(lm(reporting.boot$Final ~ reporting.boot$Group
+ reporting.boot$Baseline, reporting.boot))[2]

}

conf.int1 <- quantile(diff.mean1, c(0.025, 0.975), na.rm = TRUE)

# Fitting a linear model and calculating CIs without imputation

model2 <- lm(data2$Final ~ data2$Baseline + data2$Group)

summary(model2)

na <- 9

nb <- 9

set.seed(222222)

diff.mean2 <- c()

for (k in 1:nboot){

  sel <- sample(1:(na + nb), na + nb, rep = TRUE)  # selected articles

  reporting.boot <- data2[sel, ]

  diff.mean2[k] <- coefficients(lm(reporting.boot$Final ~ reporting.boot$Group
+ reporting.boot$Baseline, reporting.boot))[2]

}

conf.int2 <- quantile(diff.mean2, c(0.025, 0.975), na.rm = TRUE)
```

CONSORT 2010 checklist of information to include when reporting a randomised trial*

Section/Topic	Item No	Checklist item	Reported on page No
**Title and abstract**			
	1a	Identification as a randomised trial in the title	1
	1b	Structured summary of trial design, methods, results, and conclusions (for specific guidance see CONSORT for abstracts)	2
**Introduction**			
Background and objectives	2a	Scientific background and explanation of rationale	3-4
	2b	Specific objectives or hypotheses	4
**Methods**			
Trial design	3a	Description of trial design (such as parallel, factorial) including allocation ratio	5: Trial design and study setting
	3b	Important changes to methods after trial commencement (such as eligibility criteria), with reasons	10: Deviations from the protocol
Participants	4a	Eligibility criteria for participants	5: Eligibility criteria
	4b	Settings and locations where the data were collected	5: Eligibility criteria, and 9: Pilot work
Interventions	5	The interventions for each group with sufficient details to allow replication, including how and when they were actually administered	6: Interventions
Outcomes	6a	Completely defined pre-specified primary and secondary outcome measures, including how and when they were assessed	7-8:Outcomes
	6b	Any changes to trial outcomes after the trial commenced, with reasons	10: Deviations from the protocol
Sample size	7a	How sample size was determined	9: Power analysis

For peer review only - http://bmjopen.bmj.com/site/about/guidelines.xhtml

	7b	When applicable, explanation of any interim analyses and stopping guidelines	N/A
Randomisation:			
Sequence generation	8a	Method used to generate the random allocation sequence	9: Randomisation and blinding
	8b	Type of randomisation; details of any restriction (such as blocking and block size)	9: Randomisation and blinding
Allocation concealment mechanism	9	Mechanism used to implement the random allocation sequence (such as sequentially numbered containers), describing any steps taken to conceal the sequence until interventions were assigned	9: Randomisation and blinding
Implementation	10	Who generated the random allocation sequence, who enrolled participants, and who assigned participants to interventions	9: Randomisation and blinding
Blinding	11a	If done, who was blinded after assignment to interventions (for example, participants, care providers, those assessing outcomes) and how	9: Randomisation and blinding
	11b	If relevant, description of the similarity of interventions	6: Interventions
Statistical methods	12a	Statistical methods used to compare groups for primary and secondary outcomes	10: Statistical methods
	12b	Methods for additional analyses, such as subgroup analyses and adjusted analyses	10: Statistical methods
**Results**			
Participant flow (a diagram is strongly recommended)	13a	For each group, the numbers of participants who were randomly assigned, received intended treatment, and were analysed for the primary outcome	11: Results and Figure 2 (flow diagram)
	13b	For each group, losses and exclusions after randomisation, together with reasons	11: Results and Figure 2 (flow diagram)
Recruitment	14a	Dates defining the periods of recruitment and follow-up	11: Results
	14b	Why the trial ended or was stopped	N/A
Baseline data	15	A table showing baseline demographic and clinical characteristics for each group	11: Results

For peer review only - http://bmjopen.bmj.com/site/about/guidelines.xhtml

Numbers analysed	16	For each group, number of participants (denominator) included in each analysis and whether the analysis was by original assigned groups	11: Results and Figure 2 (flow diagram)
Outcomes and estimation	17a	For each primary and secondary outcome, results for each group, and the estimated effect size and its precision (such as 95% confidence interval)	11 and 12: Primary Outcome, Secondary outcome
	17b	For binary outcomes, presentation of both absolute and relative effect sizes is recommended	N/A
Ancillary analyses	18	Results of any other analyses performed, including subgroup analyses and adjusted analyses, distinguishing pre-specified from exploratory	N/A
Harms	19	All important harms or unintended effects in each group (for specific guidance see CONSORT for harms)	12: Harms
**Discussion**			
Limitations	20	Trial limitations, addressing sources of potential bias, imprecision, and, if relevant, multiplicity of analyses	13: Strengths and limitations
Generalisability	21	Generalisability (external validity, applicability) of the trial findings	13: Implications
Interpretation	22	Interpretation consistent with results, balancing benefits and harms, and considering other relevant evidence	13-14: Discussion
**Other information**			
Registration	23	Registration number and name of trial registry	5: Trial design and study setting
Protocol	24	Where the full trial protocol can be accessed, if available	5: Trial design and study setting
Funding	25	Sources of funding and other support (such as supply of drugs), role of funders	15: Funding and role of funders

For peer review only - http://bmjopen.bmj.com/site/about/guidelines.xhtml

*We strongly recommend reading this statement in conjunction with the CONSORT 2010 Explanation and Elaboration for important clarifications on all the items. If relevant, we also recommend reading CONSORT extensions for cluster randomised trials, non-inferiority and equivalence trials, non-pharmacological treatments, herbal interventions, and pragmatic trials. Additional extensions are forthcoming: for those and for up to date references relevant to this checklist, see www.consort-statement.org.

For peer review only

For peer review only - http://bmjopen.bmj.com/site/about/guidelines.xhtml

BMJ Open

Effect of an editorial intervention to improve the completeness of reporting of randomised trials: a randomised controlled trial

Journal:	*BMJ Open*
Manuscript ID	bmjopen-2020-036799.R1
Article Type:	Original research
Date Submitted by the Author:	31-Mar-2020
Complete List of Authors:	Blanco, David; Universitat Politecnica de Catalunya, Statistics and Operations Research Department; Université de Paris, CRESS, INSERM, INRA<br>Schroter, Sara; BMJ Editorial,<br>Aldcroft, Adrian<br>Moher, David; Ottawa Hospital Research Institute, Centre for Journalology, Clinical Epidemiology Program<br>Boutron, Isabelle; Université de Paris, CRESS, INSERM, INRA<br>Kirkham, Jamie J.; Manchester University, Centre for Biostatistics, Manchester Academic Health Science Centre<br>Cobo, Erik; Universitat Politecnica Catalunya, Statistics and Operational Research Department
**Primary Subject Heading**:	Medical publishing and peer review
Secondary Subject Heading:	Research methods, Evidence based practice
Keywords:	MEDICAL JOURNALISM, STATISTICS & RESEARCH METHODS, MEDICAL EDUCATION & TRAINING

SCHOLARONE™ Manuscripts

I, the Submitting Author has the right to grant and does grant on behalf of all authors of the Work (as defined in the below author licence), an exclusive licence and/or a non-exclusive licence for contributions from authors who are: i) UK Crown employees; ii) where BMJ has agreed a CC-BY licence shall apply, and/or iii) in accordance with the terms applicable for US Federal Government officers or employees acting as part of their official duties; on a worldwide, perpetual, irrevocable, royalty-free basis to BMJ Publishing Group Ltd ("BMJ") its licensees and where the relevant Journal is co-owned by BMJ to the co-owners of the Journal, to publish the Work in this journal and any other BMJ products and to exploit all rights, as set out in our licence.

The Submitting Author accepts and understands that any supply made under these terms is made by BMJ to the Submitting Author unless you are acting as an employee on behalf of your employer or a postgraduate student of an affiliated institution which is paying any applicable article publishing charge ("APC") for Open Access articles. Where the Submitting Author wishes to make the Work available on an Open Access basis (and intends to pay the relevant APC), the terms of reuse of such Open Access shall be governed by a Creative Commons licence – details of these licences and which Creative Commons licence will apply to this Work are set out in our licence referred to above.

Other than as permitted in any relevant BMJ Author's Self Archiving Policies, I confirm this Work has not been accepted for publication elsewhere, is not being considered for publication elsewhere and does not duplicate material already published. I confirm all authors consent to publication of this Work and authorise the granting of this licence.

For peer review only

Effect of an editorial intervention to improve the completeness of reporting of randomised trials: a randomised controlled trial

David Blanco[1, 2] (ORCID: 0000-0003-2961-9328), Sara Schroter[3] (ORCID: 0000-0002-8791-8564), Adrian Aldcroft[3] (ORCID: 0000-0003-0106-720X), David Moher[4] (ORCID: 0000-0003-2434-4206), Isabelle Boutron[2] (ORCID: 0000-0002-5263-6241), Jamie J Kirkham[5**](ORCID: 0000-0003-2579-9325) and Erik Cobo[1**](ORCID: 0000-0002-3534-5602).

[1]Statistics and Operations Research Department, Universitat Politècnica de Catalunya, Barcelona, Spain.

[2]Université de Paris, CRESS, INSERM, INRA, France.

[3]The BMJ, London, UK.

[4]Centre for Journalology, Clinical Epidemiology Program, Ottawa Hospital Research Institute, Ottawa, Canada.

[5]Centre for Biostatistics, Manchester Academic Health Science Centre, University of Manchester, Manchester, UK.

Correspondence to David Blanco; david.blanco.tena@upc.edu

**JJK and EC are joint senior authors.

For peer review only

For peer review only - http://bmjopen.bmj.com/site/about/guidelines.xhtml

Abstract

Objective: To evaluate the impact of an editorial intervention to improve completeness of reporting of reports of randomised trials.

Design: Randomised controlled trial (RCT).

Setting: BMJ Open's quality improvement programme.

Participants: 24 manuscripts describing RCTs.

Interventions: We used an R Shiny application to randomise manuscripts (1:1 allocation ratio, blocks of 4) to the intervention (n=12) or control group (n=12). The intervention was performed by a researcher with expertise in the content of CONsolidated Standards of Reporting Trials (CONSORT) and consisted of an evaluation of completeness of reporting of eight core CONSORT items using the submitted checklist to locate information, and the production of a report containing specific requests for authors based on the reporting issues found, provided alongside the peer review reports. The control group underwent usual peer review.

Outcomes: Primary outcome - number of adequately reported items (0-8 scale) in the revised manuscript after the first round of peer review. The main analysis was intention-to-treat (n=24) and we imputed the scores of lost to follow-up manuscripts (rejected after peer review and not resubmitted). Secondary outcome - proportion of manuscripts where each item was adequately reported. Two blinded reviewers assessed outcomes independently and in duplicate and solved disagreements by consensus. We also recorded the amount of time to perform the intervention.

Results: Manuscripts in the intervention group (mean: 7.01; SD: 1.47) were more completely reported than those in the control group (mean: 5.68; SD: 1.43); mean difference 1.43 (95% CI: 0.31 to 2.58). We observed the main differences in items 6a (Outcomes), 9 (Allocation concealment mechanism), 11a (Blinding), and 17a (Outcomes and estimation). Mean time to perform the intervention was 87 (SD 42) minutes.

For peer review only - http://bmjopen.bmj.com/site/about/guidelines.xhtml

Conclusions: We demonstrated the benefit of involving a reporting guideline expert in the editorial process. Improving the completeness of RCTs is essential for enhancing their usability.

Trial registration: *ClinicalTrials.gov*, Identifier NCT03751878.

Strengths and weaknesses

- We used a randomised controlled trial design and implemented the intervention in a real editorial context.
- Outcome assessment was blinded and in duplicate.
- We focused only on eight items of one reporting guideline (CONSORT).
- The intervention was performed in only one journal.

For peer review only

Introduction

The lack of transparency and accuracy of research reports has been pointed out as one of the main factors causing research waste (1). Adequate reporting allows researchers to replicate results, generate new hypothesis or compare the results of different studies; it allows health care professionals to make clinical decisions; it allows governments to change public policies; and it helps patients to be aware of what healthcare options they have (2).

Reporting guidelines (RGs) are sets of minimum recommendations for authors, usually in the form of a checklist, on how to report research methods and findings so that no relevant information is omitted (2). Since the inception in 1996 of the Consolidated Standards of Reporting Trials (CONSORT) for the reporting of randomised controlled trials (RCTs) (3), hundreds of RGs for different study types, data, and preclinical and clinical areas have been developed (4). CONSORT is currently one of the most well-established RGs and has been revised and updated twice (5,6).

Most RGs have not been evaluated as to whether they actually improve completeness of reporting. Even for those that have been shown to be beneficial, such as CONSORT, the degree of author adherence is poor (7). For this reason, a range of interventions aimed to improve adherence to RGs have been proposed, and the impact of some of these on completeness of reporting have been evaluated. A recent scoping review identified and classified 31 interventions targeting different stakeholders, including authors, peer reviewers, journal editors, medical schools and ethics boards (8). Among these, only four were assessed in RCTs and their effects were varied (9–12). Most of the studies included in the scoping review described observational studies that evaluated the pooled effect of different journal strategies, which ranged from making available editorial statements that endorse certain RGs, recommending or requiring authors to follow RGs in the "Instructions to authors", and requiring authors to submit a completed RG checklist together with the manuscript. However, these actions have been shown not to have the desired effect (13–16). In contrast, completeness of reporting improved remarkably when editors were in the process of checking adherence to RGs (17).

For peer review only - http://bmjopen.bmj.com/site/about/guidelines.xhtml

Recently, many biomedical journals have opted for requiring the submission of RG checklists alongside the manuscript. While sometimes checking these is delegated to peer reviewers, journal editors generally report that this task goes beyond the role of these and that it may even decrease the quality of peer-review reports (18). If checking reporting issues becomes a standard exercise for peer reviewers, some editors are afraid that peer reviewers may be less likely to comment on important aspects of a manuscript, such as its importance, novelty, and relevance. Involving trained experts or administrative staff could be a way to make the most of this editorial strategy (18).

Study objectives

We describe an RCT to evaluate the effect of an editorial intervention performed by a researcher with expertise in CONSORT on the completeness of reporting of trials submitted to BMJ Open, compared to the standard peer review process.

Methods

Trial design and study setting

This was a two-arm parallel randomised trial (1:1 allocation ratio) conducted in collaboration with BMJ Open, an open-access general medical journal (published by the BMJ Publishing Group) that requests the submission of completed CONSORT checklists for RCTs. Prior to recruitment, we registered the study in ClinicalTrials.gov with the identifier NCT03751878 and uploaded the study protocol (19).

Eligibility criteria

Manuscripts were eligible for inclusion if (i) they were original research articles reporting the results of an RCT submitted to BMJ Open, (ii) they had passed the first editorial filter and had been subsequently sent out for peer review, and (iii) authors of these manuscripts had provided a completed CONSORT checklist as part of the submission process. Apart from the standard two-arm parallel RCTs, which are covered by the standard CONSORT guidelines (20), we also included RCTs that require the use of the official CONSORT extensions for different design aspects (cluster (21), non-inferiority and equivalence (22), pragmatic (23), N-of-1 trials (24), Pilot and feasibility (25), and within person trials (26)) and intervention types (herbal

For peer review only - http://bmjopen.bmj.com/site/about/guidelines.xhtml

(27), non-pharmacologic (28), acupuncture (29) and Chinese herbal medicine formulas (30)) in all areas of clinical research. We excluded studies that claimed to be RCTs but used deterministic allocation methods and secondary trial analysis studies.

Interventions

We designed a three-step intervention based on the results of our previous work (8,18) ensuring no disruption to usual editorial procedures. The lead investigator (DB), a PhD student with a background in statistics who had worked for two years on the topic of improving adherence to RGs and who had expertise in the content of CONSORT, performed the intervention. Firstly, he assessed completeness of reporting of eight core CONSORT items (see paragraph below) using the submitted checklist to locate the information corresponding to each item. Secondly, he produced a standardised report containing precise requests to be addressed by authors. This report included a point by point description of the reporting issues found, requests to the authors to include the missing information (see example in Box 1), as well as examples extracted from the CONSORT Explanation and Elaboration document (20). Finally, DB uploaded the report to the manuscript tracking system of the journal (ScholarOne) to make it accessible to the manuscript handling editor, who included this additional report in the decision letter to authors alongside the standard peer-review reports. Manuscripts randomised to the control group underwent the usual peer review process. In Figure 1, we display a schema of the study design.

For peer review only - http://bmjopen.bmj.com/site/about/guidelines.xhtml

Please, make the following revisions:

- For CONSORT Item 8a ("*Method used to generate the random allocation sequence*"), please report the exact method you used to generate the random allocation sequence.
    - Example from CONSORT: "*Randomization sequence was created using Stata M.N (StataCorp, College Station, TX) statistical software*".
- For CONSORT Item 11a ("*If done, who was blinded after assignment to interventions and how*"), please specify in "Trial design and setting" who was blinded in the study and do not just state that it was a double-blind randomised trial.
    - Example from CONSORT: "*Whereas patients and physicians allocated to the intervention group were aware of the allocated arm, outcome assessors and data analysts were kept blinded to the allocation*"

Box 1: Example of report reflecting the reporting issues found

The intervention was focused on eight core CONSORT items (see Box 2) which are essential for researchers evaluating the risk of bias of RCTs when conducting systematic reviews (31) and which are usually poorly reported (32).

We considered an item as adequately reported if all subparts of it were adequately reported, according to the CONSORT E&E document (20) and the corresponding E&E documents for the extensions considered. For example, for CONSORT item 6a ("*Completely defined pre-specified primary and secondary outcome measures, including how and when they were assessed*"), we required the following subparts to be adequately reported: A) identified and completely defined primary and secondary outcomes, B) analysis metric and methods of aggregation for each outcome, and C) time points for each outcome.

For peer review only - http://bmjopen.bmj.com/site/about/guidelines.xhtml

Five items in the methods section:

- **Item 6a** ("*Completely defined pre-specified primary and secondary outcome measures, including how and when they were assessed*")
- **Item 8a** ("*Method used to generate the random allocation sequence*")
- **Item 9** ("*Mechanism used to implement the random allocation sequence (such as sequentially numbered containers), describing any steps taken to conceal the sequence until interventions were assigned*")
- **Item 11a** ("*If done, who was blinded after assignment to interventions (for example, participants, care providers, those assessing outcomes) and how*")
- **Item 11b** ("*If relevant, description of the similarity of interventions*")

Three items in the results section:

- **Item 13a** ("*For each group, the numbers of participants who were randomly assigned, received intended treatment, and were analysed for the primary outcome*")
- **Item 13b** ("*For each group, losses and exclusions after randomisation, together with reasons*")
- **Item 17a** ("*For each primary and secondary outcome, results for each group, and the estimated effect size and its precision (such as 95% confidence interval)*")

Box 2: Core CONSORT items considered.

The items corresponding to CONSORT extensions were assessed in addition to the standard CONSORT items. For example, we expected authors of a cluster randomised trial evaluating a pharmacologic treatment to be using the standard CONSORT checklist for all eight items and the cluster extension for items 6a, 9, 13a, 13b, and 17a. In contrast, the items requested by the Pilot and Feasibility extension substituted the standard CONSORT items, as specified in its E&E document (25). Once the recruitment had begun, we decided to discard the extension for non-pharmacologic interventions as it was not being requested by the editors, nor sent by authors.

For peer review only - http://bmjopen.bmj.com/site/about/guidelines.xhtml

In Supplementary file 1 we present further details on the rules we used to deal with not applicable items and with certain aspects of specific items.

Outcomes

- **Primary outcome**: Mean score for completeness of reporting, defined as the mean number of adequately reported items in the first revised manuscript (0 to 8 scale).
- **Secondary outcome**: Proportion of manuscripts where each item was adequately reported.

In the design phase of the study, we considered two potential scenarios where included manuscripts could potentially be lost to follow-up: (i) when editors rejected a manuscript after peer review, and (ii) when authors did not return the revised manuscript within the period requested by the handling editor after a “Minor revision” or “Major revision” editorial decision (14 and 28 days, respectively, plus, if necessary, the extra time that the editor considered appropriate). In the “Statistical methods” section, we report the methods used to impute the study outcomes for lost to follow-up articles.

Outcome evaluation was performed independently and in duplicate by two senior researchers (EC, JJK) who were blinded to manuscript allocation and had experience as authors and reviewers of RCTs. They also assessed outcomes at baseline. In cases where a manuscript was rejected after the first round of peer review, assessors could only evaluate it at baseline. However, they were not aware of the fate of that manuscript until after they had completed that evaluation. More details about the outcome assessment process can be found in Supplementary file 2.

For each of the manuscripts in the intervention group, we also recorded the amount of time it took the lead investigator to perform the intervention.

Harms

We analysed whether our intervention caused the following unintended effects: higher proportion of manuscript rejections after the first round of peer review and delays in the submission of the revised manuscripts by authors.

For peer review only - http://bmjopen.bmj.com/site/about/guidelines.xhtml

Pilot work

To inform the sample size calculation, the lead investigator assessed 12 randomly selected RCTs published in BMJ Open between April 2018 and September 2018. The proportions of adequately reported items observed in these manuscripts were used to estimate the scores for completeness of reporting of the manuscripts in the control group (usual peer review).

Furthermore, outcome assessors (EC and JJK) practised the evaluation of completeness of reporting by assessing six of the 12 RCTs mentioned above.

Power analysis

According to the assessment described in "Pilot work" section, the estimated probabilities that manuscripts in the control group adequately reported 0, 1, 2,..., and 8 items were 0, 0, 0, 0, 0, 0.17, 0.33, 0.33, and 0.17, respectively. With the intervention, we aimed to bring this distribution to 0, 0, 0, 0, 0, 0, 0, 0.5, 0.5. In other words, manuscripts in the intervention group were expected to be adequately reporting 7 or 8 items 50% of the time, respectively.

In order to relax the strong required assumptions behind using a t-test for a reduced sample size, we used bootstrapping, a simple yet powerful non-parametric technique (33). First, given the probability distributions mentioned above, we performed 10.000 simulations of the scores of *n* manuscripts. We resampled each of these simulations 10.000 times in order to calculate the 95% CI of the mean difference between groups. Finally, we calculated the study power by counting for how many of the 10.000 simulations the lower limit of this 95% CI was over 0.

Choosing a sample size of $n = 24$ manuscripts (12 per arm) and following the procedure above gave us 90% power (alpha = 0.05, two-tailed). The R code used can be found in Supplementary file 3: Script A.

Randomisation and blinding

Prior to recruitment of manuscripts, DB screened automated reports listing original research submissions to BMJ Open on ScholarOne, daily, including their ID, date of submission, title, abstract, and different parameters related to their peer review status. RCTs were identified

For peer review only - http://bmjopen.bmj.com/site/about/guidelines.xhtml

for possible inclusion based on the title and abstract and then checked against our eligibility criteria until the desired sample size was achieved.

Every time a manuscript met our eligibility criteria, DB introduced its ID into an R Shiny application (34) created by a senior statistician (JAG) (see Supplementary file 3: Script B), which randomised the manuscript to the intervention or the control group (1:1 allocation ratio, blocks of 4). Manuscripts were stratified according to whether there was an applicable CONSORT extension for that study or not. To avoid allocation bias, each ID could only be introduced once.

As part of the usual submission process, all authors are informed that BMJ Publishing Group has a quality improvement programme and their manuscript might be entered into a study. However, authors of included manuscripts were not explicitly informed that their manuscripts were part of an RCT.

Outcome assessors were blinded to allocation and to each other's evaluation. Handling editors of the included manuscripts and the investigator performing the intervention (DB) were not blinded.

Statistical methods

We carried out statistical analysis using R version 3.6.0 (35).

For the primary outcome, we adjusted a linear regression model with the baseline score of the manuscript as the only covariate. We calculated the 95% confidence interval using bootstrapping (see Supplementary file 3: Script C).

The main analysis of the primary outcome was intention-to-treat: all manuscripts were included in this analysis regardless of whether they were lost to follow-up. We imputed the scores of lost to follow-up manuscripts with a value of *8-b*, where *b* was the baseline score of the manuscript. This imputation strategy aimed to reflect the fact that rejecting RCTs of low baseline quality could be considered an editorial success. In addition, we assessed the sensitivity of the results by carrying out a complete case analysis and analysing the best case (manuscripts in the intervention group reached the maximum score and controls did not

For peer review only - http://bmjopen.bmj.com/site/about/guidelines.xhtml

improve) and worst case (manuscripts in the intervention group did not improve and controls reached the maximum score) scenarios.

We did not plan any subgroup analysis (see protocol) and so none are reported.

Deviations from the protocol

The last criterion listed above (iii) authors of the manuscripts had provided a completed CONSORT checklist) was not included in the first version of the protocol but we implemented it before recruitment started. The reason was that, despite that the submission of the CONSORT checklist for trials is mandatory, we observed that handling editors were occasionally overlooking this requirement and sending out manuscripts of trials for peer review that did not include one. Secondly, we initially used a t-test to calculate the study power and planned to use it for the primary outcome analysis. However, for the reasons described in the "Power analysis" section we used a bootstrap approach and the study power increased from the 85% stated in the protocol to 90%. Thirdly, we decided to assess the baseline scores for completeness of reporting for the included manuscripts in order to adjust for these in the primary outcome analysis. With this we tried to avoid that a difference in the baseline scores between the two groups could make the intervention seem to have a larger or smaller effect than it actually had. Finally, we added a best- and worst-case scenario analysis to assess the sensitivity of the primary outcome results.

Reporting guidelines

We report this manuscript in accordance to CONSORT 2010 (6).

Patient and public involvement

Patients were not study participants and were not involved in setting the research question, designing the study, the conduct of the study or the interpretation of the results.

For peer review only - http://bmjopen.bmj.com/site/about/guidelines.xhtml

Results

Between 31 October 2018 and 4 April 2019, we screened 62 manuscripts that described RCTs submitted to BMJ Open. Among these, we excluded 38 either because they were rejected without peer review (n = 34) or because the authors did not provide the CONSORT checklist (n = 4). We randomised the remaining 24 to the intervention (n = 12) or control (n = 12) groups. Six manuscripts (25%) were lost to follow-up (intervention n = 3, control n = 3) as they were rejected after the first round of peer review and therefore not returned to authors for revision (scenario (i) in "Outcomes" section). No manuscripts were lost to follow-up in scenario (ii) as all authors returned the revised manuscripts within the given time. Therefore, 18 manuscripts (intervention n = 9, control n = 9) were revised by authors. Figure 2 shows the flow diagram of the study.

Most manuscripts (n = 19, 79%) required at least one extension: non-pharmacologic (intervention n = 10; control n = 8), pilot and feasibility (n = 3; n = 4), cluster (n = 2; n = 1). Table 1 displays the baseline characteristics of the included manuscripts.

The mean (SD) baseline score for completeness of reporting (0 to 8 scale) prior to peer review in the intervention (n = 12) and control (n = 12) groups was 4.35 (1.88) and 4.85 (1.79), respectively. The mean (SD) baseline score of the manuscripts that later passed the first round of peer review (n = 18) were much more complete (scores almost double) than those that were rejected after the first round of peer review (n = 6): 5.23 (1.35) versus 2.68 (1.75).

Primary outcome

For the intention-to-treat analysis (n = 24), the manuscripts that received the intervention were more completely reported than the ones that underwent the standard review process: intervention group (mean: 7.01; SD: 1.47) versus control group (mean: 5.68; SD: 1.43). After adjusting for the baseline score, the mean difference in scores between the two groups was 1.43 (95% CI: 0.31 to 2.58); manuscripts in the intervention group reported on average 1.43 (out of 8) items more adequately than those receiving the standard peer review. Regarding the sensitivity analysis, for the complete case (n = 18) the mean (SD) scores for the intervention and control groups were 7.45 (1.00) and 5.90 (1.35), giving an adjusted difference of 1.75 (95% CI: 0.80 to 2.75). The best- and worst-case scenario analysis (n=24)

For peer review only - http://bmjopen.bmj.com/site/about/guidelines.xhtml

lead to adjusted differences of 2.62 (95% CI: 1.49 to 3.65) and 0.03 (95% CI: -1.45 to 1.63) respectively. Table 2 summarises these results.

Figure 3 shows the evolution of the 18 manuscripts that were revised and resubmitted. From the nine manuscripts in the intervention group, six of them achieved the maximum score and another two improved. In contrast, the only manuscript in the control group that reached the maximum score already had that score at baseline. Three manuscripts in the control group slightly improved (1, 1, and 2 points respectively). We identified that 3 out of 4 of these improvements were the result of comments made by the standard peer reviewers, rather than the authors themselves.

Secondary outcome

Figure 4 displays the proportions of manuscripts where each CONSORT item was adequately reported. We observed the main differences favouring the intervention group in items 6a (Outcomes), 9 (Allocation concealment mechanism), 11a (Blinding), and 17a (Outcomes and estimation).

Feasibility of the intervention

The mean (SD) time taken to perform the intervention was 87 (42) minutes. Supplementary file 4 displays a scatter plot that compares the amount of time spent to perform the intervention and the baseline score of the 12 manuscripts in the intervention group. There was no correlation between these two variables (ρ = 0.08).

Harms

We did not identify any unintended effects. There were no differences between the intervention and the control groups for the proportion of manuscripts that were rejected after the first round of peer review (3 of 12, 25%, for each group). Furthermore, all authors submitted the revised manuscripts within the period requested by the handling editor.

Discussion

We found that the introduction during the peer review process of an editorial intervention performed by a researcher with expertise in the content of CONSORT significantly improved

For peer review only - http://bmjopen.bmj.com/site/about/guidelines.xhtml

the completeness of reporting of trials submitted to BMJ Open compared to standard peer review. Six of the nine manuscripts in the intervention group achieved the maximum score and another two improved. In contrast, the only manuscript in the control group with the maximum score at follow-up already had reached that score at baseline. We observed the main differences favouring the intervention group in items 6a (Outcomes), 9 (Allocation concealment mechanism), 11a (Blinding), and 17a (Outcomes and estimation). Moreover, providing authors with extra comments on reporting issues did not seem to discourage them from revising the manuscript as all authors returned the revised manuscripts within the standard 28 days requirement.

Strengths and limitations

This study has several strengths: the randomised trial design; the fact that the intervention was performed in a real editorial context alongside peer review reports with no disruption to usual editorial procedures; and the fact that the outcome assessment process was blinded and in duplicate.

We also note some limitations that affect the generalisability of our results. Our intervention was focused only on CONSORT, which is one of the most well-established RGs. It could potentially be more difficult for authors to fully address reviewers' comments about other less familiar RGs. We only included one journal and the same effect might not be observed in other journals. Nonetheless, we purposefully selected a very large general medical journal receiving international submissions across multiple specialties. We considered only eight core CONSORT items that are essential for evaluating the risk of bias of RCTs and not the whole checklist.

Implications

Given the importance of improving the completeness of reporting of randomised trials and given the ineffectiveness of the strategies that biomedical journals are currently implementing (13–16), it is time to take a step forward. Our study provides empirical evidence of the effectiveness of involving in the peer review process a researcher with expertise in CONSORT. In this study, the intervention was carried out by a PhD student and was implemented alongside peer review. However, this intervention could potentially be done by

For peer review only - http://bmjopen.bmj.com/site/about/guidelines.xhtml

For peer review only

trained editorial staff, editors or external consultants. The demonstrated benefits of our intervention should encourage journal editors to find the best way to make this feasible.

We note that the complete-case analysis and the best-case scenario of the sensitivity analysis point to a larger effect of the intervention than the main analysis. The worst-case scenario shows no effect. However, this scenario would assume that (1) the three rejected manuscripts in the intervention group would not improve from baseline; and that (2) all manuscripts in the control group would reach the maximum score. This scenario seems highly unlikely given that 8 out of 9 manuscripts that were not rejected in the intervention group improved from baseline and that only three controls improved and none of these reached the maximum score.

More than two decades ago, scientists started to discuss the importance of including statistical reviews as part of the publication process (36). Nowadays, statistical reviews have become widespread among top medical journals. These are usually performed by a statistician and focus on the methodological and statistical aspects of the study. As methodological issues are often not fixable, statistical reviews are key to determining the fate of manuscripts and preventing unsound research getting published (37). Completeness of reporting reviews should also become a key component in the publication system. As reporting issues are often improvable, these reviews should not generally aim to determine whether a manuscript should be published or not, but to improve their transparency. This would both help editors and peer reviewers make decisions on the manuscripts and improve the usability of published papers.

A few other RCTs have assessed different strategies for improving adherence to RGs. A recent RCT did not show that requesting authors to submit a checklist improves completeness of reporting and called for more stringent editorial policies (16). The implementation of a writing aid tool for authors (COBWEB) led to a moderate improvement in the completeness of reporting (11) whereas getting a statistician to perform an additional review against RGs showed a slightly positive but smaller than hypothesised effect (10). Suggesting peer reviewers to check RGs (9) and implementing the web-based tool WebCONSORT at the manuscript revision stage showed no positive impact (12). However, comparisons between

For peer review only - http://bmjopen.bmj.com/site/about/guidelines.xhtml

the results of our study and these RCTs must be made with caution as they targeted different RGs and were carried out in different settings.

The time taken for us to perform the intervention (87 minutes on average, with great variation between manuscripts) is clearly a barrier to wider implementation. Future research could evaluate whether this intervention should be focused on the whole CONSORT checklist, which would make this strategy even more time-consuming, or only on a few core items (such as those we found to be poorly reported). Also, it would be interesting to assess whether similar benefits can be obtained for other widely used RGs, such as SPIRIT (38) or PRISMA (39). Furthermore, this intervention could also be tested at other points in the editorial process, for example before the first decision is made on the manuscript or between the first decision and the invitation of external peer reviewers. For this study, we discarded both options for pragmatic reasons, as we did not want to alter the usual editorial process. While the first could be too resource intensive for journals, the latter would imply the same effort and the manuscript would undergo more transparent and accurate peer review, which could make the task of peer reviewers and handling editors easier and more efficient. We strongly recommend that journals always carry out experiments in real editorial contexts, such as this study, before considering making any changes in their policies.

Conclusions

This study provides evidence that involving a researcher with expertise in CONSORT in the process of evaluating RG checklists submitted by authors significantly improves the completeness of reporting of randomised trials. This is essential to reducing the research waste associated with inadequate reporting of RCT methods and findings. Journal editors should consider revising their peer review processes to find ways to make this intervention workable, tailoring it to their preferences.

For peer review only - http://bmjopen.bmj.com/site/about/guidelines.xhtml

Acknowledgements

We thank the MiRoR Project and Marie Sklodowska-Curie Actions for their support. This RCT is the third part of DB's PhD project whose first part was a scoping review to identify and classify interventions to improve adherence to RGs (8) and whose second part was a survey to explore biomedical editors' opinion on various editorial interventions to improve adherence to RGs (18).

We thank BMJ Open for collaborating with this project, and also José Antonio González and Jordi Cortés (Universitat Politècnica de Catalunya) for collaborating in the process of developing the R codes used to perform the randomisation and outcome analysis.

Funding and role of funders

This study is part of the ESR 14 research project from the Methods in Research on Research (MiRoR) project (http://miror-ejd.eu/), which has received funding from the European Union's Horizon 2020 research and innovation programme under the Marie Sklodowska-Curie grant agreement No 676207. DM is supported through a University Research Chair (University of Ottawa). The sponsor and the funding source had no role in the design of this study and will not have any role during its execution, analyses, interpretation of the data, or decision to submit results.

Contributor roles

DB: Conceptualisation, Methodology, Software, formal analysis, Investigation, writing – original draft preparation

SS: Conceptualisation, Methodology, Resources, Writing – review and editing

AA: Conceptualisation, Methodology, Resources, Writing – review and editing

DM: Conceptualisation, Methodology, Writing – review and editing

IB: Conceptualisation, Methodology, Writing – review and editing

JJK: Conceptualisation, Methodology, Outcome evaluation; Writing – review and editing, Supervision

EC: Conceptualisation, Methodology, Outcome evaluation; Writing – review and editing, Supervision

Ethics approval and informed consent

For peer review only - http://bmjopen.bmj.com/site/about/guidelines.xhtml

We obtained ethics approval from the Research Committee of the Governing Council of the Universitat Politècnica de Catalunya (UPC). Ref: EC 02. Date: 13 September 2018.

We did not seek consent from authors as this was part of a quality improvement programme at BMJ Open. However, all authors of submitted manuscripts are routinely informed that BMJ has a research programme and that they can opt out if they wish.

Data management and confidentiality

All data related to the study were collated and managed from a password protected spreadsheet file stored in a BMJ Google Drive folder.

For DB to access BMJ Open's manuscript tracking system, a confidentiality agreement with BMJ Publishing Group was signed to certify that BMJ Publishing group wished to disclose information to DB and that DB wished to receive this information on a confidential basis.

Data sharing

The content of the intervention reports will appear as part of the peer review history of the manuscripts included in the study. However, in order to protect confidentiality, we are not releasing any dataset including individual manuscript data or outcome data identifying the performance of individual participants.

Declaration of interests

AA is Editor in Chief of BMJ Open. AA was involved in the design of the study and writing the manuscript but not in data collection or data analysis. AA was not involved in the decision-making on this manuscript; the handling editor for the manuscript was instructed to raise any queries to the Deputy Editor and AA was blinded to the editorial notes and discussion of the manuscript. The editorial team were instructed not to treat this manuscript any differently and that they should reject it if the reviewers felt it was not methodologically robust.

SS is Senior Researcher at The BMJ. DM is Director of the Canadian EQUATOR Centre. IB is deputy director of French EQUATOR Centre. DM, IB, and EC are members of the CONSORT steering group.

Transparency statement

For peer review only - http://bmjopen.bmj.com/site/about/guidelines.xhtml

The lead author affirms that this manuscript is an honest, accurate, and transparent account of the study being reported; that no important aspects of the study have been omitted; and that any discrepancies from the study as planned (and, if relevant, registered) have been explained.

For peer review only

For peer review only - http://bmjopen.bmj.com/site/about/guidelines.xhtml

Figures and tables

Figure 1: Schema of the study design.

Figure 2: CONSORT flow diagram.

Figure 3: Evolution of the scores for all manuscripts that passed the first round of peer review (n=18).

Figure 4: Proportion of manuscripts (n=18) where each CONSORT item is adequately reported. **Legend**: Cont: control group; Int: intervention group. CONSORT items:

- 6a ("*Completely defined pre-specified primary and secondary outcome measures, including how and when they were assessed*")
- 8a ("*Method used to generate the random allocation sequence*")
- 9 ("*Mechanism used to implement the random allocation sequence (such as sequentially numbered containers), describing any steps taken to conceal the sequence until interventions were assigned*")
- 11a ("*If done, who was blinded after assignment to interventions (for example, participants, care providers, those assessing outcomes) and how*")
- 11b ("*If relevant, description of the similarity of interventions*")
- 13a ("*For each group, the numbers of participants who were randomly assigned, received intended treatment, and were analysed for the primary outcome*")
- 13b ("*For each group, losses and exclusions after randomisation, together with reasons*")
- 17a ("*For each primary and secondary outcome, results for each group, and the estimated effect size and its precision (such as 95% confidence interval)*")

For peer review only

For peer review only - http://bmjopen.bmj.com/site/about/guidelines.xhtml

Table 1: Baseline characteristics of the included RCTs.

			Intervention (n=12)	Control (n=12)
Study Design	Standard parallel-group		7 (58%)	7 (58%)
	Cluster		2 (17%)	1 (8%)
	Pilot & feasibility		3 (25%)	4 (33%)
Type of intervention	Pharmacologic		2 (17%)	4 (33%)
	Non-pharmacologic		10 (83%)	8 (67%)
		Behavioural	4 (33%)	3 (25%)
		E-health & tele-health strategies	3 (25%)	2 (17%)
		Medical devices	2 (17%)	1 (8%)
		Surgery	0 (0%)	1 (8%)
		Others	1 (8%)	1 (8%)
Single- or multi-centre	Single-centre		8 (67%)	5 (42%)
	Multi-centre		4 (33%)	7 (58%)
Number of participants	≤ 50		5 (42%)	2 (17%)
	> 50 & ≤ 100		3 (25%)	7 (58%)
	> 100		4 (33%)	3 (25%)
Registered in a trial registry	Yes		11 (92%)	11 (92%)
	No		1 (8%)	1 (8%)

For peer review only

For peer review only - http://bmjopen.bmj.com/site/about/guidelines.xhtml

First author's affiliation	Asia	3 (25%)	3 (25%)
	UK	3 (25%)	5 (42%)
	Europe	2 (17%)	3 (25%)
	USA	2 (17%)	0 (0%)
	Australia	2 (17%)	0 (0%)
	Brazil	0 (0%)	1 (8%)
Sponsorship	Investigator-initiated	12 (100%)	10 (83%)
	Industry-initiated	0 (0%)	2 (17%)

For peer review only

Table 2: Scores for completeness of reporting scores in the control and intervention groups.

Outcome	Intervention group Mean (SD)		Control group Mean (SD)		Mean difference in final scores* (95% CI)
	**Baseline**	**Final**	**Baseline**	**Final**	
Completeness of reporting (0 to 8 scale) with imputation (n = 24)	4.35 (1.88)	7.01 (1.47)	4.85 (1.79)	5.68 (1.43)	1.43 (0.31 to 2.58)
Completeness of reporting (0 to 8 scale) without imputation (complete case analysis, n = 18)	5.01 (1.32)	7.45 (1.00)	5.46 (1.41)	5.90 (1.35)	1.75 (0.80 to 2.75)
Completeness of reporting (0 to 8 scale) in the best-case scenario (n=24)	4.35 (1.88)	7.59 (0.89)	4.85 (1.79)	5.18 (1.89)	2.62 (1.49 to 3.65)
Completeness of reporting (0 to 8 scale) in the worst-case scenario (n=24)		6.18 (2.61)		6.43 (1.49)	0.03 (-1.45 to 1.63)

*Adjusted for baseline score.

For peer review only

Supplementary files

Supplementary file 1: Rules for the evaluation of completeness of reporting.

Supplementary file 2: Further details of the outcome assessment process.

Supplementary file 3: R scripts.

Supplementary file 4: Scatter plot of the amount of time spent to perform the intervention and the baseline score of the 12 manuscripts in the intervention group.

For peer review only - http://bmjopen.bmj.com/site/about/guidelines.xhtml

References

1. Glasziou P, Altman DG, Bossuyt P, Boutron I, Clarke M, Julious S, et al. Reducing waste from incomplete or unusable reports of biomedical research. Lancet. 2014 Jan 18;383(9913):267–76.

2. EQUATOR Network. What is a reporting guideline? [Internet]. 2019 [cited 2019 Nov 28]. Available from: http://www.equator-network.org/about-us/what-is-a-reporting-guideline/

3. Begg C, Cho M, Eastwood S, Horton R, Moher D, Olkin I, et al. Improving the quality of reporting of randomized controlled trials. The CONSORT statement. JAMA. 1996 Aug 28;276(8):637–9.

4. EQUATOR Network. Library for health research reporting [Internet]. 2019 [cited 2019 Dec 2]. Available from: https://www.equator-network.org/library/

5. Altman DG, Schulz KF, Moher D, Egger M, Davidoff F, Elbourne D, et al. The revised CONSORT statement for reporting randomized trials: Explanation and elaboration. Ann Intern Med. 2001 Apr 17;134(8):663–94.

6. Schulz KF, Altman DG, Moher D, CONSORT Group. CONSORT 2010 statement: updated guidelines for reporting parallel group randomised trials. BMJ. 2010 Mar 23;340:c332.

7. Samaan Z, Mbuagbaw L, Kosa D, Borg Debono V, Dillenburg R, Zhang S, et al. A systematic scoping review of adherence to reporting guidelines in health care literature. J Multidiscip Healthc. 2013 May;6:169–88.

8. Blanco D, Altman D, Moher D, Boutron I, Kirkham JJ, Cobo E. Scoping review on interventions to improve adherence to reporting guidelines in health research. BMJ Open. 2019;9(5).

9. Cobo E, Selva-O'Callagham A, Ribera JM, Cardellach F, Dominguez R, Vilardell M. Statistical reviewers improve reporting in biomedical articles: A randomized trial. PLoS One. 2007;2(3):e332.

10. Cobo E, Cortés J, Ribera JM, Cardellach F, Selva-O'Callaghan A, Kostov B, et al. Effect of using reporting guidelines during peer review on quality of final manuscripts submitted to a biomedical journal: masked randomised trial. BMJ. 2011 Nov 22;343(nov22 2):1084.

11. Barnes C, Boutron I, Giraudeau B, Porcher R, Altman DG, Ravaud P. Impact of an online

For peer review only - http://bmjopen.bmj.com/site/about/guidelines.xhtml

writing aid tool for writing a randomized trial report: the COBWEB (Consort-based WEB tool) randomized controlled trial. BMC Med. 2015 Sep 15;13(1):221.

12. Hopewell S, Boutron I, Altman DG, Barbour G, Moher D, Montori V, et al. Impact of a web-based tool (WebCONSORT) to improve the reporting of randomised trials: results of a randomised controlled trial. BMC Med. 2016 Nov 28;14(1):199.

13. Turner L, Shamseer L, Altman DG, Schulz KF, Moher D. Does use of the CONSORT Statement impact the completeness of reporting of randomised controlled trials published in medical journals? A Cochrane review. Syst Rev. 2012 Nov 29;1(1):60.

14. Stevens A, Shamseer L, Weinstein E, Yazdi F, Turner L, Thielman J, et al. Relation of completeness of reporting of health research to journals' endorsement of reporting guidelines: systematic review. BMJ. 2014 Jun 25;348(jun25 2):g3804–g3804.

15. Blanco D, Biggane AM, Cobo E. Are CONSORT checklists submitted by authors adequately reflecting what information is actually reported in published papers? Trials. 2018 Dec 29;19(1):80.

16. Hair K, Macleod MR, Sena ES. A randomised controlled trial of an Intervention to Improve Compliance with the ARRIVE guidelines (IICARus). Res Integr Peer Rev. 2019 Dec;4(1).

17. Pandis N, Shamseer L, Kokich VG, Fleming PS, Moher D. Active implementation strategy of CONSORT adherence by a dental specialty journal improved randomized clinical trial reporting. J Clin Epidemiol. 2014 Sep;67(9):1044–8.

18. Blanco D, Hren D, Kirkham JJ, Cobo E, Schroter S. A survey exploring biomedical editors' perceptions of editorial interventions to improve adherence to reporting guidelines. F1000Research. 2019 Sep 24;8:1682.

19. Blanco D, Schroter S, Moher D, Aldcroft A, Boutron I, Kirkham JJ, et al. Evaluating the Impact of Assessing During Peer Review the CONSORT Checklist Submitted by Authors [Internet]. 2019 [cited 2019 Nov 28]. Available from: https://clinicaltrials.gov/ct2/show/NCT03751878

20. Moher D, Hopewell S, Schulz KF, Montori V, Gøtzsche PC, Devereaux PJ, et al. CONSORT 2010 explanation and elaboration: updated guidelines for reporting parallel group randomised trials. BMJ. 2010;340.

21. Campbell MK, Piaggio G, Elbourne DR, Altman DG, CONSORT Group. Consort 2010 statement:

For peer review only - http://bmjopen.bmj.com/site/about/guidelines.xhtml

extension to cluster randomised trials. BMJ. 2012 Sep 4;345:e5661.

22. Piaggio G, Elbourne DR, Pocock SJ, Evans SJW, Altman DG. Reporting of noninferiority and equivalence randomized trials: Extension of the CONSORT 2010 statement. Vol. 308, JAMA - Journal of the American Medical Association. 2012. p. 2594–604.

23. Zwarenstein M, Treweek S, Gagnier JJ, Altman DG, Tunis S, Haynes B, et al. Improving the reporting of pragmatic trials: an extension of the CONSORT statement. BMJ. 2008;337(nov11 2):a2390.

24. Vohra S, Shamseer L, Sampson M, Bukutu C, Schmid CH, Tate R, et al. CONSORT extension for reporting N-of-1 trials (CENT) 2015 Statement. J Clin Epidemiol. 2016;76:9–17.

25. Eldridge SM, Chan CL, Campbell MJM, Bond CM, Hopewell S, Thabane L, et al. CONSORT 2010 statement: Extension to randomised pilot and feasibility trials. BMJ. 2016;355.

26. Pandis N, Chung B, Scherer RW, Elbourne D, Altman DG. CONSORT 2010 statement: Extension checklist for reporting within person randomised trials. BMJ. 2017;357.

27. Gagnier JJ, Boon H, Rochon P, Moher D, Barnes J, Bombardier C. Reporting Randomized, Controlled Trials of Herbal Interventions: An Elaborated CONSORT Statement. Ann Intern Med. 2013 Mar 7;144(5):364.

28. Boutron I, Moher D, Altman DG, Schulz KF, Ravaud P. Extending the CONSORT Statement to Randomized Trials of Nonpharmacologic Treatment : Explanation and Elaboration. Ann Intern Med. 2008;148(4):295–309.

29. MacPherson H, Altman DG, Hammerschlag R, Youping L, Taixiang W, White A, et al. Revised STandards for Reporting Interventions in Clinical Trials of Acupuncture (STRICTA): Extending the CONSORT Statement. PLoS Med. 2010 Jun 8;7(6):e1000261.

30. Cheng C, Wu T, Shang H, Li Y, Altman DG, Moher D, et al. CONSORT Extension for Chinese Herbal Medicine Formulas 2017: Recommendations, Explanation, and Elaboration. Ann Intern Med. 2018;167(2):112.

31. Chauvin A, Moher D, Altman D, Schriger DL, Alam S, Hopewell S, et al. A protocol of a cross-sectional study evaluating an online tool for early career peer reviewers assessing reports of randomised controlled trials. BMJ Open. 2017 Sep 15;7(9):e017462.

For peer review only - http://bmjopen.bmj.com/site/about/guidelines.xhtml

32. Hopewell S, Collins GS, Boutron I, Yu L-M, Cook J, Shanyinde M, et al. Impact of peer review on reports of randomised trials published in open peer review journals: retrospective before and after study. BMJ. 2014;349.

33. Efron B. Bootstrap Methods: Another Look at the Jackknife. Ann Stat. 1979 Jan;7(1):1–26.

34. González J-A. Randomisation of BMJ Open manuscripts [Internet]. 2019 [cited 2019 Dec 29]. Available from: http://shiny-eio.upc.edu/MIRoR/randomassign/

35. R Core Team. R: A language and environment for statistical computing [Internet]. 2019. Available from: http://www.r-project.org/

36. Altman DG. Statistical reviewing for medical journals. Vol. 17, Ltd. Statistics in Medicine Statist. Med. John Wiley & Sons; 1998.

37. Petrovečki M. The role of statistical reviewer in biomedical scientific journal. Biochem Medica. 2009;19(3).

38. Chan A-W, Tetzlaff JM, Altman DG, Laupacis A, Gøtzsche PC, Krleža-Jerić K, et al. SPIRIT 2013 statement: Defining standard protocol items for clinical trials. Ann Intern Med. 2013;158(3):200–7.

39. Moher D, Liberati A, Tetzlaff J, Altman DG. Preferred reporting items for systematic reviews and meta-analyses: The PRISMA statement. Vol. 339, BMJ (Online). 2009. p. 332–6.

For peer review only

For peer review only - http://bmjopen.bmj.com/site/about/guidelines.xhtml

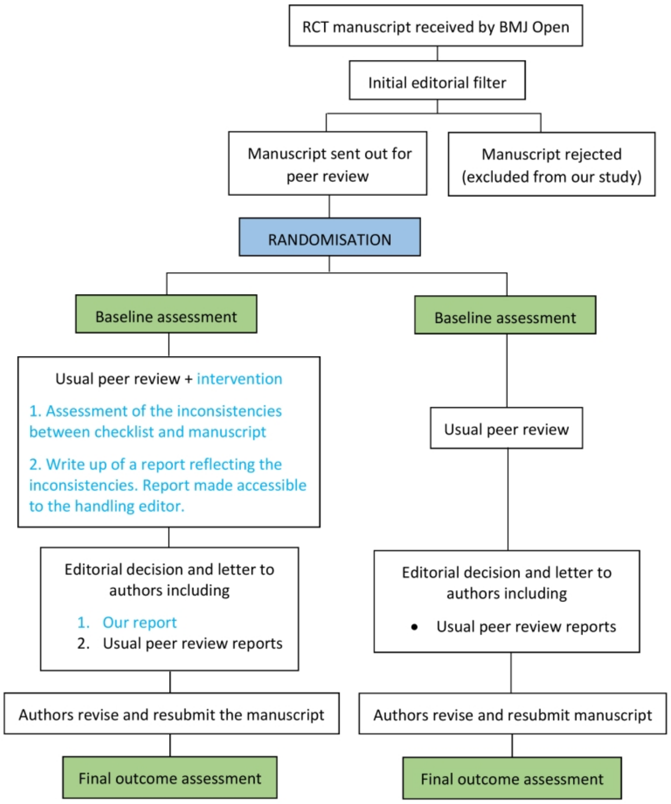

Schema of the study design

209x297mm (300 x 300 DPI)

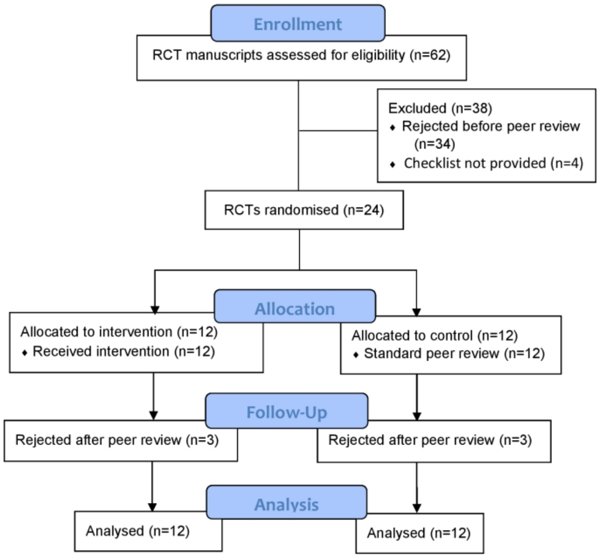

CONSORT flow diagram

209x297mm (300 x 300 DPI)

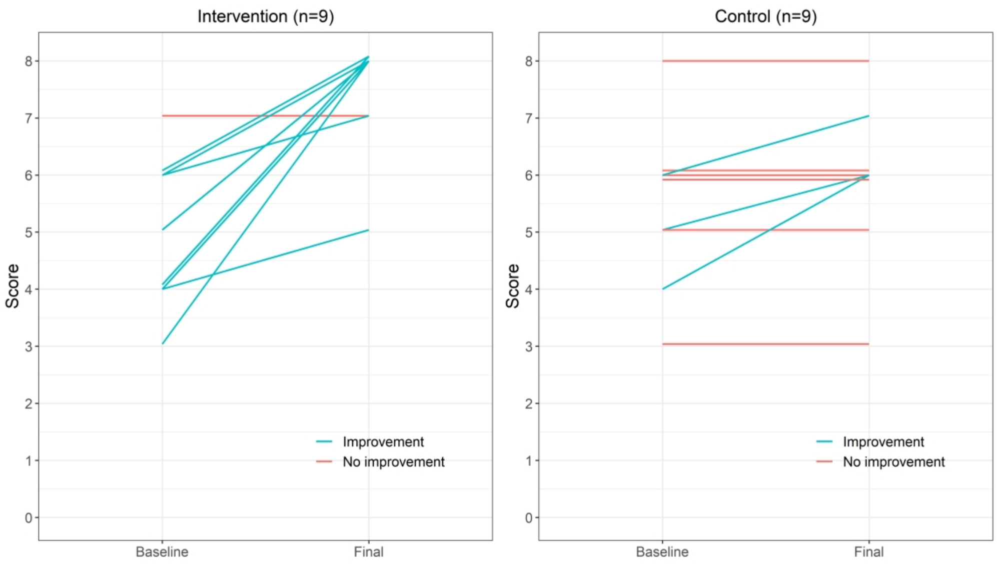

Evolution of the scores for all manuscripts that passed the first round of peer review (n=18).

304x177mm (300 x 300 DPI)

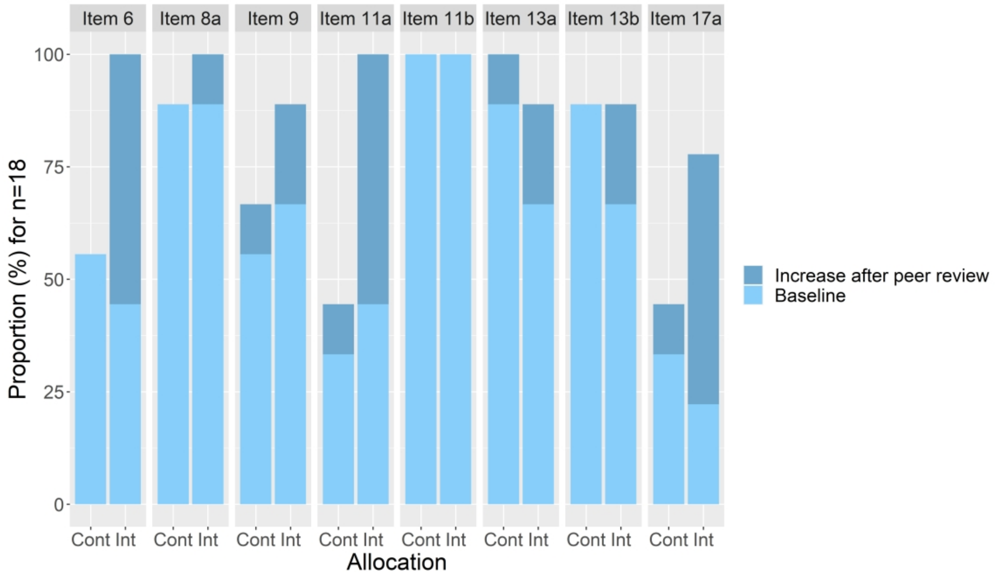

Proportion of manuscripts (n=18) where each CONSORT item is adequately reported. Legend: Cont: control group; Int: intervention group. CONSORT items:

• 6a ("Completely defined pre-specified primary and secondary outcome measures, including how and when they were assessed")
• 8a ("Method used to generate the random allocation sequence")
• 9 ("Mechanism used to implement the random allocation sequence (such as sequentially numbered containers), describing any steps taken to conceal the sequence until interventions were assigned")
• 11a ("If done, who was blinded after assignment to interventions (for example, participants, care providers, those assessing outcomes) and how")
• 11b ("If relevant, description of the similarity of interventions")
• 13a ("For each group, the numbers of participants who were randomly assigned, received intended treatment, and were analysed for the primary outcome")
• 13b ("For each group, losses and exclusions after randomisation, together with reasons")
• 17a ("For each primary and secondary outcome, results for each group, and the estimated effect size and its precision (such as 95% confidence interval)")

304x177mm (300 x 300 DPI)

N/As for a certain item

For items reported as N/A in the CONSORT checklist, we consider them as:

- Adequately reported if (i) the item did not apply and therefore it did not have to be reported, and (ii) the item applied and it was actually reported although the page number was not given.
- Inadequately reported if the item did apply but it was not adequately reported.

Rules about specific items:

- **Item 8a** ("*Method used to generate the random allocation sequence*"): inadequately reported if authors have reported this information elsewhere but not in the main body of the article. According to CONSORT, "*it is important that information on the process of randomisation is included in the body of the main article and not as a separate supplementary file; where it can be missed by the reader*".
- **Item 11a** ("*If done, who was blinded after assignment to interventions (for example, participants, care providers, those assessing outcomes) and how*"): adequately reported if blinding was not performed and authors explicitly said so, and inadequately reported if blinding was assumed to be not performed and authors did not mention it in the manuscript.
- **Item 13a** ("*For each group, the numbers of participants who were randomly assigned, received intended treatment and were analysed for the primary outcome*") and **item 13b** ("*For each group, losses and exclusions after randomisation, together with reasons*")**:** the corresponding information could be included either in the text or in the flow diagram. If information was only included in the discussion, it was considered as inadequately reported.
- **Item 17a** ("*For each primary and secondary outcome, results for each group, and the estimated effect size and its precision (such as 95% confidence interval)*")**:** adequately reported if there was a correspondence between the outcomes in the results section and the ones listed in the methods section (and therefore evaluated in Item 6a).
- **Extension of Item 17a** for Pilot and Feasibility trials ("*For each objective, results including expressions of uncertainty (such as 95% confidence interval) for any estimates. If relevant, these results should be by randomised group*"): we did not expect authors to

For peer review only

report the effect sizes but the results (plus expressions of uncertainty) for each objective.

For peer review only

We divided the 24 included manuscripts into 4 batches of 6 manuscripts.

Every time DB detected in the submissions report (see "Preliminary work" section) that all 6 manuscripts of each batch had been revised by authors, he first made available to the outcome assessors the submitted version of the manuscript (version 1). Assessors had to complete the evaluation form for each manuscript independently and in duplicate. This form included the CONSORT extensions to be used. Assessors could explicitly indicate in it that they wanted to discuss a specific item with the other assessor. Once they were done with all manuscripts' version 1, DB informed them of the discrepancies between their evaluations, which were resolved by consensus. Afterwards, he shared the manuscript revised by the authors (version 2) and we repeated the outcome evaluation process.

This process was done for the 4 batches of 6 manuscripts.

For peer review only

Script A: Power calculation

```
na <- 12
nb <- 12
A <- 0:8
B <- 0:8
#Estimated probabilities of reporting 0,...,8 items in the intervention group (a) and control group (b)
pra <- c(0, 0, 0, 0, 0, 0, 0, 5, 5)
pra <- pra/sum(pra)
prb <- c(0, 0, 0, 0, 0, 2, 4, 4, 2)
prb <- prb/sum(prb)
nboot <- 1000
N <- 1000
#Matrices containing random samples of scores for the two groups
ma <- matrix(sample(A, pr = pra, replace = TRUE, siz = N * na), ncol = N)
mb <- matrix(sample(B, pr = prb, replace = TRUE, siz = N * nb), ncol = N)
#Generation of N confidence intervals
sign1 <- c()
for (i in 1:N) {
  reporting <- data.frame(
    score = c(ma[, i], mb[, i]),
    group = factor(rep(c("Control", "Intervention"), c(na, nb)))
  )
  # Bootstrapping
  diff.mean1 <- c()
  for (k in 1:nboot){
    sel <- sample(1:(na + nb), na + nb, rep = TRUE)  # selected articles
    reporting.boot <- reporting[sel, ]
    diff.mean1[k]<- with(reporting.boot, diff(tapply(score, group, mean)))
  }
  conf.int1 <- quantile(diff.mean1, c(0.025, 0.975))
  sign1[i] <- conf.int1[2] < 0 #Checking if the CI crosses 0
}
power <- sum(sign1)/N
```

Script B: Randomisation of manuscripts (R Shiny application)

```
#File 1
Sys.setlocale("LC_ALL", "es_ES.UTF-8") #to be sure that accents in text will be allowed in plots
library(shiny)
library(shinyalert)
fluidPage(
  useShinyalert(),
  fluidRow(
        headerPanel('Randomisation of BMJ Open manuscripts'),
        wellPanel(
                textInput("identif", "Please enter the manuscript ID:", width='33%'),
                textAreaInput("titulo", "Please enter the manuscript title, or at least its first words:"),
                selectInput("tipo", "Does it correspond to an extension of CONSORT?", choices=c("", "Yes", "No"), width='33%'),
                actionButton("send", "SUBMIT")
        ),
        p() ,
        wellPanel(
                #h3('Data'),
                #p("(just for testing purpose)"),
                #tableOutput("asig"),
                #actionButton("borrar", "RESET")
        )
  )
```

```
#File 2
library(shiny)
library(shinyalert)
library(blockrand)
is.void = function(x) {
   if (is.null(x)) return(TRUE)
         x == ''
}
shinyServer(function(input, output, session) {
upda = reactiveValues(asg=NA)
archi = "asignacion.dat"
```

```
#File 2 (continuation)

if (file.exists(archi)) {

        upda$asg = read.table(archi, header=TRUE, sep='\t', stringsAsFactors=FALSE)

} else {

   upda$asg = data.frame()

}

set.seed(6374422)

ext <- blockrand(n=100, id.prefix='E', block.prefix='B',stratum='Extension', block.sizes=c(2,2))

noext <- blockrand(n=100, id.prefix='N', block.prefix='B',stratum='No Ext.', block.sizes=c(2,2))

  go <- eventReactive(input$send, {

   list(id=input$identif, tit=input$titulo, ex=input$tipo)

  })

block_random = function(tip) {

  if (dim(upda$asg)[1]>0) {

    X = subset(upda$asg, strat==tip)

         x = dim(X)[1]

  } else x = 0

  if (tip=="Yes") {

                g = ext$treatment[x+1]

        } else if (tip=="No") {

                g = noext$treatment[x+1]

        } else return(-1)    # Error

  ifelse(g=='A', 0, 1)

}

        observeEvent(input$send, {

                Q = go()

                if (is.null(Q) | is.void(Q)) {

                        shinyalert(title="Please fill in the input.", type="error", showConfirmButton=TRUE, confirmButtonText="OK", timer=0)

                        return()

                }

                id = Q$id

                check.id = grep("^bmjopen-201[89]-[0-9]{6}$", id)

                if (length(check.id) == 0) {

                        shinyalert(title="Wrong ID.", text="Please enter a valid BMJ code.", type="error", showConfirmButton=TRUE, confirmButtonText="OK", timer=0)

return()

                }
```

```
#File 2 (continuation)

id = Q$id

        check.id = grep("^bmjopen-201[89]-[0-9]{6}$", id)

        if (length(check.id) == 0) {

            shinyalert(title="Wrong ID.", text="Please enter a valid BMJ code.", type="error", showConfirmButton=TRUE, confirmButtonText="OK", timer=0)

            return()

        }

        if (Q$ex=='') {

            shinyalert(title="Empty field:", text="extension of CONSORT?", type="error", showConfirmButton=TRUE, confirmButtonText="OK", timer=0)

            return()

        }

        if (Q$tit=='') {

            shinyalert(title="Empty field:", text="please provide a title.", type="error", showConfirmButton=TRUE, confirmButtonText="OK", timer=0)

            return()

        }

        n = dim(upda$asg)[1]

        if (n>0) {

            I = which(upda$asg$id==id)

            if (length(I)>0) {

                shinyalert(title="Invalid ID:", text="this ID has been already assigned.", type="error", showConfirmButton=TRUE, confirmButtonText="OK", timer=0)

                return()

            }

        }

        txt = paste("Go on with the manuscript '<i>", Q$tit, "</i>', with ID <b>",Q$id, "</b>, which <b>", ifelse(Q$ex=='Yes', 'corresponds', 'does not correspond'), "</b> to an extension of CONSORT:", sep='')

        shinyalert(title='Confirm inclusion', text=txt, closeOnEsc=TRUE, closeOnClickOutside=FALSE, html=TRUE, type="warning", showConfirmButton=TRUE, showCancelButton=TRUE, confirmButtonText="Right, go on", cancelButtonText="NO, stop", timer=0, imageUrl="", callbackR = Success)

    })

    Success = function(x) if (x != FALSE) {

        Q = go()

        g = block_random(Q$ex)

        if (g == -1) return

        L = list(id=Q$id, title=Q$tit, strat=Q$ex, group=g, date=date())

        upda$asg = rbind(upda$asg, as.data.frame(L))

        write.table(upda$asg, archi, sep='\t', row.names=FALSE)
```

```
#File 2 (continuation)

                    filename = tempfile()

                    interv = ifelse(g==0, "CONTROL (0)", "INTERVENTION (1)")

                    cat(sprintf("Manuscript ID: %s\nTitle: %s\nExtension of CONSORT: %s\nAssigned to: %s\n",

                              Q$id, Q$tit, Q$ex, interv), file=filename)

                    # preparar y mandar mensaje

                    dest = 'david.blanco@hotmail.com'

                    Msg = tempfile()

                    comm = paste('echo "To:', dest, '\nFrom: jose.a.gonzalez@upc.edu\nSubject: A manuscript has been assigned\n"| (cat -', filename, ') >', Msg)

                    system(comm)

                    system(paste("ssmtp", dest, "<", Msg))


                    updateTextInput(session, "identif", "Please enter the manuscript ID:", value='')

                    updateTextAreaInput(session, "titulo", "Please enter the manuscript title, or at least its first words:", value='')

                    updateSelectInput(session, "tipo", "Does it correspond to an extension of CONSORT?", choices=c("", "Yes", "No"))

          }


          observeEvent(input$borrar, {

                    shinyalert(title='Are you sure?', text="This action will remove the assignations.", closeOnEsc=TRUE, closeOnClickOutside=FALSE, html=TRUE, type="warning", showConfirmButton=TRUE, showCancelButton=TRUE, confirmButtonText="Yes, remove them", cancelButtonText="NO, don't reset", timer=0, imageUrl="", animation=TRUE, callbackR = function(x) { if(x != FALSE) { upda$asg = data.frame(); file.remove(archi) } })

          })

          output$asig = renderTable( {

                    upda$asg

          } )

})
```

Script C: Primary outcome analysis

```
# Loading the data
data <- read.csv2('Scores.txt', header = TRUE, sep = '')
data2 <- subset(data, data$Imputation==0)
# Fitting a linear model and calculating CIs with imputation
model1 <- lm(data$Final ~ data$Baseline + data$Group)
summary(model1)
na <- 12
nb <- 12
nboot <- 10000
set.seed(111111)
diff.mean1 <- c()
for (k in 1:nboot){
  sel <- sample(1:(na + nb), na + nb, rep=TRUE)  # selected articles
  reporting.boot <- data[sel, ]
  diff.mean1[k] <- coefficients(lm(reporting.boot$Final ~ reporting.boot$Group
+ reporting.boot$Baseline, reporting.boot))[2]
}
conf.int1 <- quantile(diff.mean1, c(0.025, 0.975), na.rm = TRUE)
# Fitting a linear model and calculating CIs without imputation
model2 <- lm(data2$Final ~ data2$Baseline + data2$Group)
summary(model2)
na <- 9
nb <- 9
set.seed(222222)
diff.mean2 <- c()
for (k in 1:nboot){
  sel <- sample(1:(na + nb), na + nb, rep = TRUE)  # selected articles
  reporting.boot <- data2[sel, ]
  diff.mean2[k] <- coefficients(lm(reporting.boot$Final ~ reporting.boot$Group
+ reporting.boot$Baseline, reporting.boot))[2]
}
conf.int2 <- quantile(diff.mean2, c(0.025, 0.975), na.rm = TRUE)
```

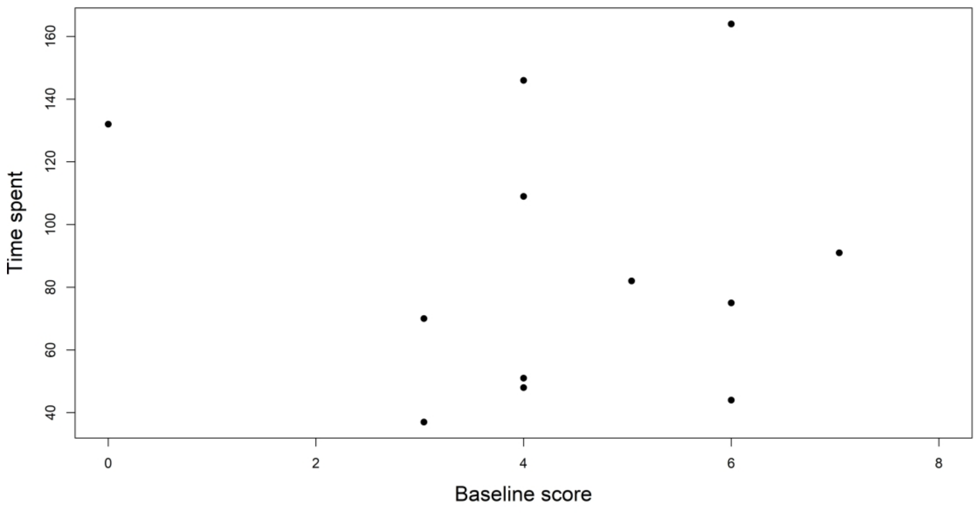

304x177mm (300 x 300 DPI)

CONSORT 2010 checklist of information to include when reporting a randomised trial*

Section/Topic	Item No	Checklist item	Reported on page No
**Title and abstract**			
	1a	Identification as a randomised trial in the title	1
	1b	Structured summary of trial design, methods, results, and conclusions (for specific guidance see CONSORT for abstracts)	2
**Introduction**			
Background and objectives	2a	Scientific background and explanation of rationale	3-4
	2b	Specific objectives or hypotheses	4
**Methods**			
Trial design	3a	Description of trial design (such as parallel, factorial) including allocation ratio	5: Trial design and study setting
	3b	Important changes to methods after trial commencement (such as eligibility criteria), with reasons	10: Deviations from the protocol
Participants	4a	Eligibility criteria for participants	5: Eligibility criteria
	4b	Settings and locations where the data were collected	5: Eligibility criteria, and 9: Pilot work
Interventions	5	The interventions for each group with sufficient details to allow replication, including how and when they were actually administered	6: Interventions
Outcomes	6a	Completely defined pre-specified primary and secondary outcome measures, including how and when they were assessed	7-8:Outcomes
	6b	Any changes to trial outcomes after the trial commenced, with reasons	10: Deviations from the protocol
Sample size	7a	How sample size was determined	9: Power analysis

For peer review only

	7b	When applicable, explanation of any interim analyses and stopping guidelines	N/A
Randomisation:			
Sequence generation	8a	Method used to generate the random allocation sequence	9: Randomisation and blinding
	8b	Type of randomisation; details of any restriction (such as blocking and block size)	9: Randomisation and blinding
Allocation concealment mechanism	9	Mechanism used to implement the random allocation sequence (such as sequentially numbered containers), describing any steps taken to conceal the sequence until interventions were assigned	9: Randomisation and blinding
Implementation	10	Who generated the random allocation sequence, who enrolled participants, and who assigned participants to interventions	9: Randomisation and blinding
Blinding	11a	If done, who was blinded after assignment to interventions (for example, participants, care providers, those assessing outcomes) and how	9: Randomisation and blinding
	11b	If relevant, description of the similarity of interventions	6: Interventions
Statistical methods	12a	Statistical methods used to compare groups for primary and secondary outcomes	10: Statistical methods
	12b	Methods for additional analyses, such as subgroup analyses and adjusted analyses	10: Statistical methods
**Results**			
Participant flow (a diagram is strongly recommended)	13a	For each group, the numbers of participants who were randomly assigned, received intended treatment, and were analysed for the primary outcome	11: Results and Figure 2 (flow diagram)
	13b	For each group, losses and exclusions after randomisation, together with reasons	11: Results and Figure 2 (flow diagram)
Recruitment	14a	Dates defining the periods of recruitment and follow-up	11: Results
	14b	Why the trial ended or was stopped	N/A
Baseline data	15	A table showing baseline demographic and clinical characteristics for each group	11: Results

For peer review only - http://bmjopen.bmj.com/site/about/guidelines.xhtml

Numbers analysed	16	For each group, number of participants (denominator) included in each analysis and whether the analysis was by original assigned groups	11: Results and Figure 2 (flow diagram)
Outcomes and estimation	17a	For each primary and secondary outcome, results for each group, and the estimated effect size and its precision (such as 95% confidence interval)	11 and 12: Primary Outcome, Secondary outcome
	17b	For binary outcomes, presentation of both absolute and relative effect sizes is recommended	N/A
Ancillary analyses	18	Results of any other analyses performed, including subgroup analyses and adjusted analyses, distinguishing pre-specified from exploratory	N/A
Harms	19	All important harms or unintended effects in each group (for specific guidance see CONSORT for harms)	12: Harms
**Discussion**			
Limitations	20	Trial limitations, addressing sources of potential bias, imprecision, and, if relevant, multiplicity of analyses	13: Strengths and limitations
Generalisability	21	Generalisability (external validity, applicability) of the trial findings	13: Implications
Interpretation	22	Interpretation consistent with results, balancing benefits and harms, and considering other relevant evidence	13-14: Discussion
**Other information**			
Registration	23	Registration number and name of trial registry	5: Trial design and study setting
Protocol	24	Where the full trial protocol can be accessed, if available	5: Trial design and study setting
Funding	25	Sources of funding and other support (such as supply of drugs), role of funders	15: Funding and role of funders

For peer review only

*We strongly recommend reading this statement in conjunction with the CONSORT 2010 Explanation and Elaboration for important clarifications on all the items. If relevant, we also recommend reading CONSORT extensions for cluster randomised trials, non-inferiority and equivalence trials, non-pharmacological treatments, herbal interventions, and pragmatic trials. Additional extensions are forthcoming: for those and for up to date references relevant to this checklist, see www.consort-statement.org.

For peer review only
